# Automated analysis of feline posture using deep learning and geometric modeling

**DOI:** 10.3389/fvets.2026.1881772

**Published:** 2026-09-01

**Authors:** Carlos Eduardo Bezerra, Cauê Bittencourt, Renalvo Alves, Thiago Ribeiro, María Azorín-Ortuño, Gloria Nejar, Alba Barreras, Claudine Zemirline, Thomas Lewiner, Anahita Le Bourdiec, Gaëlle Pagny, Adriano Barbosa, Xu Yang, Krerley Oliveira, Thales Vieira

**Affiliations:** 1 Institute of Computing, Federal University of Alagoas (UFAL), Maceió, Brazil; 2 Isoquimen, Barcelona, Spain; 3 Ceva Santé Animale, Libourne, France; 4 Faculty of Exact Sciences and Technology, Federal University of Grande Dourados (UFGD), Dourados, Brazil; 5 Institute of Mathematics, Federal University of Alagoas (UFAL), Maceió, Brazil

**Keywords:** action recognition, computer vision, deep learning, feline behavior, posture analysis

## Abstract

**Introduction:**

Body posture provides clinically relevant information in cats, but behavioral interpretation can be challenging because postural signs are often subtle, context-dependent, and subject to inter-observer variability. We developed and evaluated a non-invasive computer vision framework for extracting clinically interpretable feline posture descriptors from side-view RGB images acquired using low-cost cameras, with the aim of supporting behavioral assessment, welfare monitoring, and future clinical decision support.

**Methods:**

The proposed framework combines object detection, anatomical segmentation, lightweight convolutional neural networks for posture and side-view classification, pose estimation, and rule-based geometric analysis within a structured processing pipeline. From standing side-view frames, the system generates descriptors including overall posture, head pitch, back inclination, back curvature, and tail configuration.

**Results:**

In image-based evaluation, the lightweight neural networks achieved 92.6% accuracy for side-view classification and 93.1% accuracy for overall posture classification while using approximately 38 times fewer trainable parameters than VGG19-based baselines. After image quality and viewpoint filtering, descriptor accuracy reached 88% for head pitch, 95% for back inclination, 90% for back curvature, and 95.47% for tail configuration. In end-to-end video experiments, frame retention after filtering depended strongly on recording conditions, particularly side-view visibility and occlusion. Nevertheless, retained frames still enabled high-accuracy estimation of head pitch, back inclination, and tail configuration.

**Discussion:**

These findings demonstrate that automated posture analysis from standard RGB video can generate structured and clinically interpretable feline body-language descriptors relevant to behavioral medicine and animal welfare assessment. Further prospective validation against established pain, stress, and welfare assessment instruments is required before clinical or diagnostic application.

## 1 Introduction

In feline medicine and welfare assessment, body posture is a clinically relevant behavioral signal, yet objective and scalable interpretation remains difficult in routine practice (1, 2). Low-cost RGB camera systems provide a practical opportunity for non-invasive behavioral monitoring in domestic and clinical environments (3). However, identifying meaningful postural patterns or behavioral anomalies still relies largely on human observation, which is time-consuming, labor-intensive, and subject to inter-observer variability.

Automatically generating detailed posture-related descriptors continuously over time can support veterinarians, veterinary nurses, and animal behavior researchers, while also enabling downstream welfare monitoring applications. These subtle physical cues are often linked to pain, stress, neurological conditions, or emotional states, and may otherwise go unnoticed in casual observation. For example, an abnormal tail position or a persistent head tilt can serve as early indicators of discomfort or illness (4). In addition, automation supports large-scale behavioral studies by providing consistent and objective data on feline body language (5). Beyond reducing human effort, such systems can significantly improve the speed, precision, and scalability of behavioral analysis, unlocking new possibilities for practical applications and scientific research. However, current approaches to animal behavior monitoring rarely address detailed, automated recognition of feline postures, and even fewer provide continuous, quantitative descriptors of body configuration over time.

In feline behavioral medicine, posture interpretation is particularly challenging due to the subtle and ambiguous nature of cats' expressive signals. Unlike more vocal species, cats often mask vulnerability through minimal postural changes, such as slight ear flattening or tail twitches, which may indicate pain, stress, or emotional state depending on context (6). Although validated ethograms provide standardized terminology and descriptors to improve objectivity, their application in routine veterinary practice remains limited (7). Accurate ethogram scoring requires prolonged observation, specialized training, and consistent interpretation, making assessments highly dependent on observer expertise and leading to inter-rater variability and potential misclassification of behavioral states.

These challenges are further amplified in busy clinical and domestic environments, where time constraints, stress-induced behavioral alterations, and multitasking hinder careful posture assessment (8). Even trained veterinary professionals and nurses may struggle to consistently detect nuanced posture-based indicators without structured decision-support tools. As a result, despite their recognized value, feline ethograms are underutilized in daily practice, highlighting the need for automated systems capable of generating continuous, objective, and reproducible posture descriptors that can complement clinical expertise and reduce subjectivity. In routine consultations, hospitalization, and home monitoring, sustained structured observation is often impractical; consequently, subtle posture-derived indicators are underused in day-to-day decision-making.

**Figure 1:**
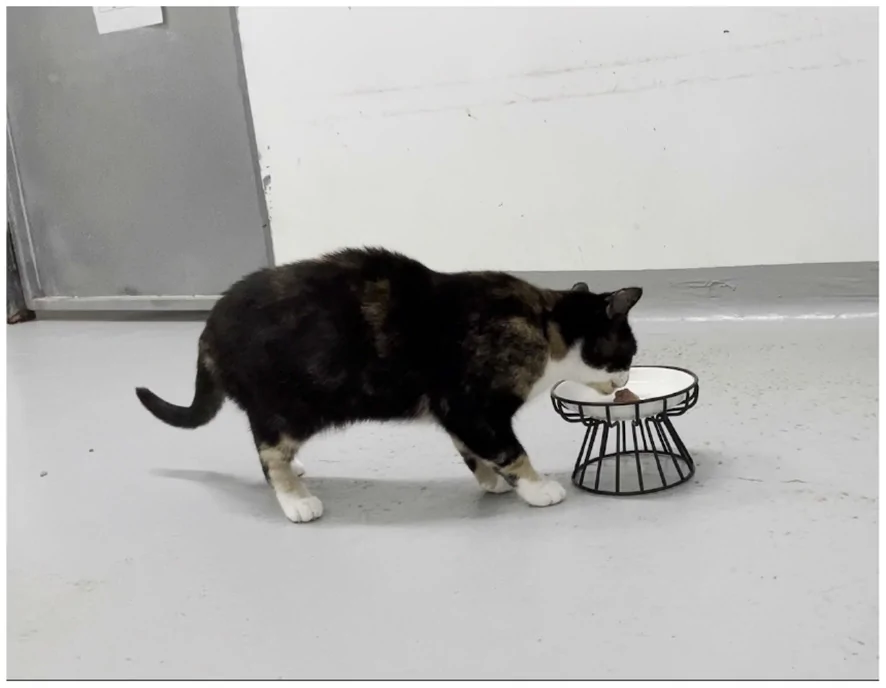
Example of an image capturing the cat's side view.

To address these challenges, we propose a novel methodology that automates cat posture analysis without manual intervention. Our approach leverages side-view images captured by a low-cost RGB camera positioned in areas frequently visited by the cat. Side views are particularly suitable for this task, as they provide a clear profile of the animal's posture, as illustrated in Figure 1.

The choice of the side-view configuration was not arbitrary. It was defined in consultation with animal behavior specialists, who indicated that lateral observation is the most informative perspective for evaluating feline posture in routine practice. Clinically relevant indicators such as trunk curvature, head pitch, and tail orientation are primarily expressed in the sagittal plane and are more reliably assessed when the full body profile is visible. Frontal or oblique views tend to introduce occlusions and depth ambiguities that complicate both human interpretation and geometric modeling. Therefore, the adoption of side-view imagery aligns the proposed computational framework with established professional assessment practices while ensuring anatomical interpretability and methodological robustness. This perspective also reflects practical ethogram-based observation workflows, where sagittal-plane features are typically the most consistently interpretable.

We propose an automated framework that integrates deep learning-based detection, pose estimation, and rule-based geometric analysis through a structured processing pipeline. The method identifies key anatomical features and infers posture descriptors such as back curvature and tail orientation. It leverages established models: YOLO (9) for object detection, DensePose (10) for anatomical part segmentation, and MMPose (11) for keypoint estimation. These are complemented by lightweight neural network classifiers and geometric rule-based algorithms that provide accurate and interpretable posture classification. Experimental results validate the effectiveness of our method, underscoring its potential for use in behavioral monitoring and animal welfare applications.

In summary, the novelty of the proposed fully automated framework stems from five main contributions: (i) a multi-model detection pipeline for robust feline keypoint estimation; (ii) lightweight task-specific neural networks for side-view validation and overall posture classification; (iii) novel geometric algorithms for silhouette- and keypoint-based posture analysis; (iv) an extensive experimental evaluation demonstrating high accuracy across all stages of the proposed approach; and (v) a clinically interpretable descriptor set aligned with feline behavioral assessment needs in veterinary and welfare contexts.

By converting routine RGB video into standardized posture descriptors, this framework can improve reproducibility of feline behavioral assessment across clinics, shelters, and research environments.

It is important to note that the scope of the present study is restricted to the accurate estimation of posture-related descriptors, and not to the assessment of welfare, pain, or emotional states. The descriptors produced by the proposed framework, namely overall posture, head pitch, back inclination, back curvature, and tail configuration, are geometric quantities validated against human-annotated ground truth; they do not, by themselves, constitute indicators of any clinical or affective condition. Establishing a link between these descriptors and clinically relevant outcomes would require a dedicated study correlating them with validated pain, stress, and welfare assessment instruments (Section 8).

The remainder of this paper is organized as follows. Section 2 reviews related work. Section 3 provides an overview of the proposed methodology, including its underlying principles and implementation steps. Section 4 describes the architectures of the overall posture classifier and the side-view classifier, while Section 5 details the geometric feature extraction process for posture analysis. Section 6 presents the high-level posture classification, introducing the head pitch, back, and tail classifiers. Section 7 reports and discusses the experimental results, emphasizing the effectiveness and limitations of the proposed approach. Finally, Section 8 concludes the paper and outlines directions for future research.

## 2 Related work

Recent advances in artificial intelligence, especially in deep learning and computer vision, have enabled a wide range of applications in animal behavior research (12, 13). In this section, we review some representative studies that illustrate the use of these techniques, progressing from general applications involving animals to more specific studies on cats, and finally to work related to feline postures.

### 2.1 Deep learning in animal behavior analysis

Numerous studies have effectively applied deep learning models to detect, monitor, and analyze animal behavior and physiology. For instance, Oliveira et al. (14) provided a comprehensive review of recent advances in deep learning-based computer vision systems for livestock science, highlighting their value in predicting phenotypes to support farm management and breeding decisions. Saoud et al. (12) continued this line of work by discussing the applications of deep learning techniques in animal behavior research, focusing on tracking, pose estimation, and behavioral analysis, while also identifying emerging trends and future challenges in the field. In addition, Kyriazakis et al. (15) demonstrated that deep learning object-detection models, including YOLOv5, can support automated video-based monitoring of clinically relevant post-weaning behavioral and posture changes in pigs under practical farm conditions. Finally, Xu et al. (16) presented a diverse wild mammal behavior dataset and introduced Efficient Dual-stream Neural Networks (EDNN), a spatiotemporal deep learning model that achieved high recognition accuracy and demonstrated an efficient, generalizable approach for automated animal behavior analysis. Although these studies demonstrate the growing impact of deep learning in behavioral research, they primarily address general or non-feline species and do not focus on detailed, posture-level analysis.

Several studies have employed models closely related to those used in our work. Ranc et al. (17) used images captured by unmanned aerial vehicles (UAVs) to detect and count deer populations, implementing three versions of the YOLO architecture alongside the Single Shot Multibox Detector (SSD) (18). Similarly, Dias (19) proposed a computer vision pipeline that combines YOLOv4 with a linear regression model to estimate the weight of croakers in fish farms. Their approach also identified feeding periods by analyzing spatial fish groupings in images, thereby reducing manual monitoring efforts. Pereira (20) explored sensory imprinting strategies in piglets, using continuous video recording and heatmap analysis to assess post-weaning feeding behavior, and demonstrated that piglets exposed to familiar sensory cues exhibited improved feed intake patterns. In another study, Zhang et al. (21) developed a method to recognize sheep behavior through spatiotemporal video analysis, combining YOLOv5, AlphaPose, and graph neural networks. This approach enabled contact-free detection of key behaviors and contributed to health monitoring in semi-housed farming systems. Finally, an interesting generalization of the YOLO model was presented by Kang et al. (22), further illustrating the versatility of modern object detection frameworks in animal-related computer vision tasks. While these studies successfully apply YOLO-based architectures to various species and contexts, none address fine-grained feline posture estimation or combine detection, segmentation, and keypoint analysis as proposed in our work.

### 2.2 Feline recognition and behavioral analysis

Beyond general animal research, a growing number of studies have focused specifically on feline behavior using computer vision and machine learning. One active line of research addresses cat identification and emotion recognition. Lin and Kuo (23) proposed a noninvasive cat face recognition method to help identify missing pets, achieving 94.1% accuracy using Convolutional Neural Networks (CNNs) for facial part detection and Support Vector Machines (SVMs) for classification based on features from 1,500 images of 150 cats. Similarly, Chen et al. (24) developed a method for recognizing and identifying individual cats using a combination of Autoencoders and CNNs, providing valuable insights into robust cat recognition models and serving as a reference point for fine-grained animal identification. Vishwakarma et al. (25) introduced a hybrid system that combines CNNs with Canny edge detection to classify cat facial expressions into basic emotional categories. Akbar et al. (26) tackled the challenge of monitoring feral cats in Australia, a major threat to native wildlife, where camera traps often capture partial or obscured views of animals. To address this, they developed a deep learning approach based on body-part identification, training ResNet-50 models on four anatomical regions (flank, back leg, front leg, and tail) using a curated dataset of 1,644 images from 10 individual cats. In the same feline-computer-vision direction, Martvel et al. (27) presented an automated landmark-based framework for cat facial analysis with applications including pain-related inference. Additional studies, such as those by Yang et al. (28) and Martvel et al. (29), further explore feline identification through deep learning.

Another important research direction involves tracking feline behaviors such as eating and drinking. Eagan et al. (30) introduced BeRSTID, a system that employs visual markers on collars and low-cost cameras to automatically monitor feeding and drinking activities of cats in shelters. Similarly, Nakajima et al. (31) fine-tuned a YOLO model on a small annotated video dataset of domestic cats and demonstrated that, even with fewer than 100 training images, individual tracking performance improved significantly. Additionally, other studies have focused on distinguishing between cats and dogs, such as (32–34), which explore interspecies image classification using various machine learning techniques.

While these studies demonstrate significant progress in feline recognition, emotion, and behavior analysis, they primarily focus on facial or part-based identification. In contrast, posture-based assessment may offer practical advantages for routine behavioral monitoring in veterinary and domestic settings. Postural cues such as crouching, tail lashing, ear positioning, and piloerection integrate information across multiple body regions and remain observable at a distance, under variable lighting conditions, and during free movement. In contrast, facial signals, although informative in controlled or close-range settings, are often subtle, breed-dependent, and sensitive to viewing angle, motion, and handling stress, which limits their applicability in everyday clinical and domestic environments. As a result, posture-based assessment tends to exhibit higher robustness and inter-rater agreement in real-world contexts, making it particularly suitable for automated, continuous monitoring systems (4).

Building on this body of research, our study prioritizes full-body feline posture analysis, integrating detection, segmentation, and geometric modeling to derive robust behavioral descriptors that are better suited to real-world welfare monitoring.

### 2.3 Posture estimation and analysis

One of the most important research directions in this field is the identification and classification of cat postures. Before discussing results specific to cats, several studies on human posture estimation provide valuable methodological insights (35–38). For instance, (39) developed a posture-based tracking scheme for multiple fish that accurately identified and tracked individual head and tail positions, recorded movement trajectories, and enabled detailed analysis of collective behaviors with minimal error. In veterinary applications, Phelan et al. (40) further highlighted the potential of computer vision and pose-based analysis for objective locomotion assessment in canines.

Focusing specifically on felines, Sui et al. (41) aimed to improve human-cat emotional interaction by recognizing and classifying aggressive behaviors in kittens during their socialization stage (3-7 weeks old). Unlike studies emphasizing basic emotional categories, this work investigated the mechanisms of offensive and defensive aggression through posture analysis. Using YOLOv5 and three classical image classification models (VGG16, ResNet50, and MobileNetV2), the authors compared performance across three aggressive postures, ultimately deploying the lightweight MobileNetV2 model as a mobile application for real-time behavioral recognition. Similarly, Fawzy et al. (42) proposed DeepCat, a deep learning-based system designed to interpret cat body language by detecting key features such as the tail, eyes, and mouth. Trained on a dataset of 10,000 automatically labeled cat images, DeepCat enables pet owners to better understand their cats' emotional states, and it is deployed as a Flutter-based mobile application for real-time use in domestic environments.

Beyond vision-based systems, some studies have explored non-wearable sensing mechanisms for animal posture analysis. For example, Pons et al. (43) developed a depth-based tracking system that detects animal body parts and postures, comparing multiple classifiers under supervised and knowledge-based paradigms. Their results demonstrated that depth tracking could be a viable alternative to wearable sensors for animal behavior monitoring. However, despite these advances, no previous work has provided a detailed, keypoint-based analysis of feline posture using a multi-model deep learning pipeline. To address this gap, we propose an automated system that integrates YOLO for object detection, DensePose for anatomical segmentation, and MMPose for keypoint estimation. This framework enables fine-grained posture interpretation from side-view images, offering a novel perspective for understanding feline behavior and contributing a new tool for veterinary behavioral monitoring.

## 3 Method overview

**Figure 2:**
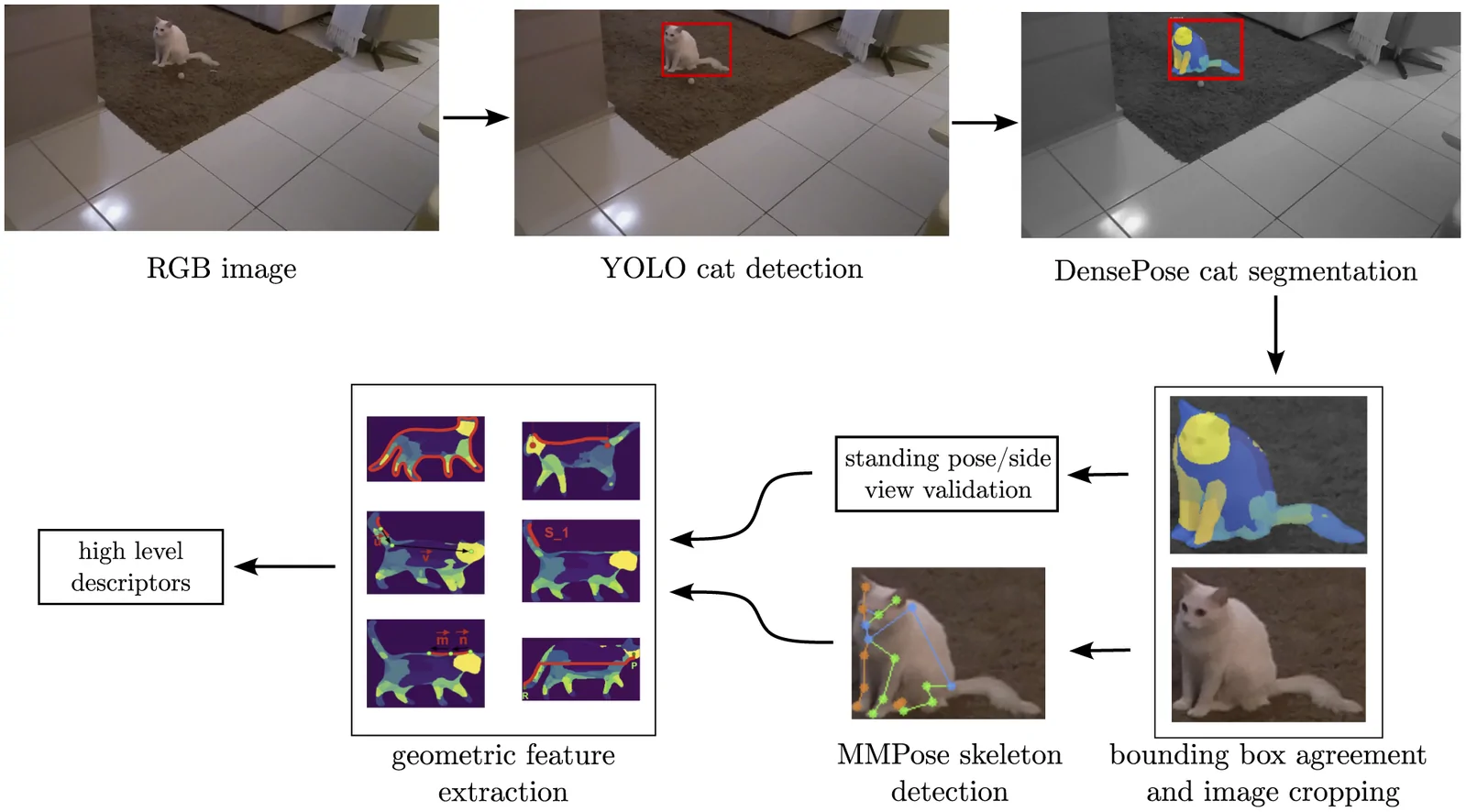
Overview of the proposed framework for automated feline posture analysis and its multi-stage processing pipeline. An input RGB frame is first processed by YOLO for cat detection and by DensePose for anatomical segmentation. After validating detection agreement and cropping the region of interest, lightweight neural classifiers are applied to confirm standing posture and side-view orientation. Frames that satisfy these conditions are then processed by MMPose for skeletal keypoint estimation. Validated keypoints and silhouettes are used for geometric feature extraction, and rule-based classifiers subsequently infer high-level posture descriptors. For clarity, some intermediate filtering and consistency checks are omitted.

This work presents a computer vision framework for automated feline posture analysis from images captured by low-cost RGB cameras. The use of low-cost cameras was motivated by the intended applicability of the framework in routine domestic, shelter, and clinical environments, where affordability and ease of deployment are important practical constraints. Figure 2 summarizes the framework architecture and its multi-stage processing pipeline. The framework outputs a set of clinically interpretable high-level posture descriptors, including:

- cat presence in the image: present or absent;
- overall posture: lying down, sitting, crouching, standing or other;
- head pitch: down or up;
- back curvature: arched or non-arched;
- back inclination: negative, neutral or positive;
- tail configuration: down, neutral or up.

By delivering structured and interpretable data, the proposed approach enables non-invasive, continuous monitoring of feline behavior, supporting early diagnosis, long-term behavioral analysis, and improved animal care across both domestic and clinical settings (44).

The image and video data analyzed in this study were derived from a previously approved palatability study in dogs and/or cats conducted by Isoquimen under animal experimentation project authorization no. 10416, reviewed and approved by the Animal Experimentation Commission of the Government of Catalonia (Comissió d'Experimentació Animal, Generalitat de Catalunya).

The resulting image and video dataset comprised more than 30 individual, non-pedigree domestic shorthair cats (*Felis catus*) with diverse coat colors and patterns. Although this provides some degree of visual diversity in terms of pelage, all data were collected at a single research facility under controlled palatability-testing conditions, which is acknowledged as a limitation to the generalizability of the framework.

**Figure 3:**
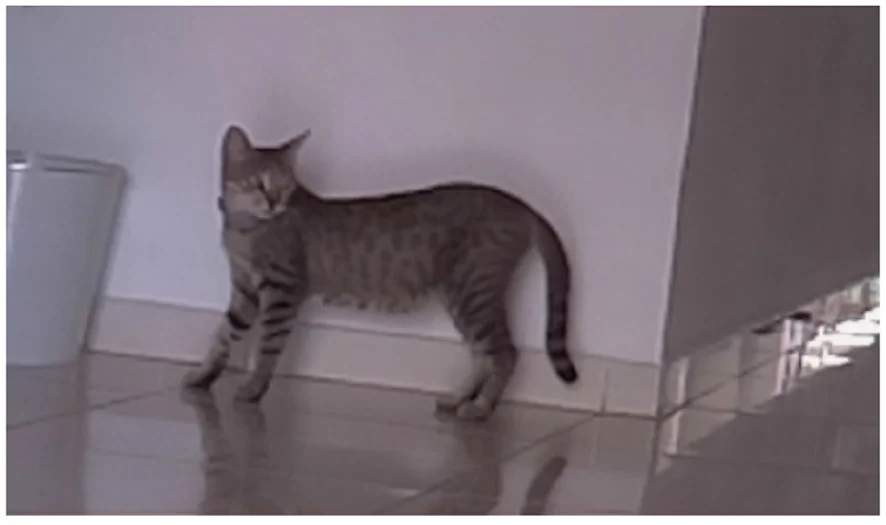
Side-view image of a cat, suitable for cat posture analysis.

We use images recorded with a low-cost RGB camera as input, capable of capturing images with a resolution of at least 1280 × 720 pixels. To enable the analysis of features such as the head, back, and tail of the cat, we analyzed only images that provide a side view of the animal. For this reason, it is necessary to choose a location for placing the camera such that the observable area in the captured images is a location the cat frequently passes through; and the images capture a side view of the cat, as illustrated in Figure 3.

**Figure 4:**
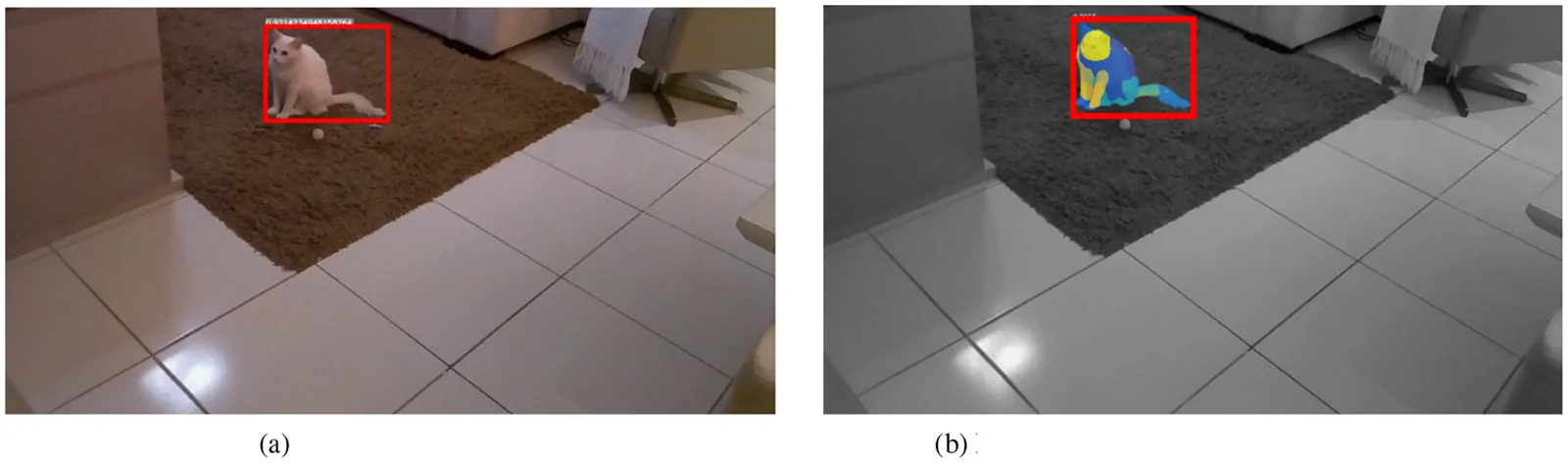
Example of cat detection and DensePose estimation, illustrating a case where the bounding boxes produced by both models coincide. The bounding boxes are depicted in red, and DensePose anatomical segments are color-coded. **(a)** YOLO bounding box. **(b)** DensePose segmentation and bounding box.

Video recordings are processed frame by frame as described in Figure 2. Given an input frame, we first detect cats in it using a YOLOv5 model (9), pre-trained on the COCO dataset (45), which includes a class for cat detection. Frames where YOLO detects one or more cats are subsequently processed with a variant of the DensePose model (46). DensePose was originally designed to map each pixel of a detected human figure to specific anatomical regions (e.g., head, back, limbs). Sanakoyeu et al. (10) extended this approach by fine-tuning DensePose on datasets containing animals with similar morphology, such as felines. In this work, we employed the DensePose architecture based on Faster R-CNN with a Feature Pyramid Network (FPN) and a ResNet-50 backbone, trained on the AP-10K dataset (47). The model configuration is available in the official DensePose repository.^1^ These models, however, are not exclusively specialized for cats and may inadvertently detect other species. To mitigate this, we combine the detection outputs of YOLO and DensePose, ensuring that pose estimation is only applied to regions confidently identified as cats, as illustrated in Figure 4.

We assess the agreement between YOLO and DensePose detections by comparing the spatial proximity of the bounding boxes generated by each model. Let *c*_*y*_ be the center of a bounding box detected by YOLO, and *c*_*d*_ be the center of a bounding box corresponding to an animal detected by DensePose. If the Euclidean distance between these centers satisfies $∥c_{y}-c_{d}∥<T_{bbox}$ for any pair of bounding boxes, the models are considered to agree on the presence of a cat in that location, and posture analysis proceeds. Otherwise, the frame is discarded. In this work, we use $T_{bbox}=50$ pixels.

For frames where the models agree, the process returns an RGB image cropped according to YOLO's bounding box, along with the corresponding cropped segmented image from DensePose (lower right rectangle in Figure 2). In the segmented output, each pixel is encoded with an integer representing an anatomical part, which is color-coded in Figure 4b for visualization purposes only.

Next, the overall posture of the cat is classified by a lightweight Convolutional Neural Network (CNN) that analyzes the DensePose image, assigning one of five classes: lying down, sitting, crouching, standing, or other. This CNN was designed and trained from scratch in this work, and is detailed in Section 4.

To estimate the remaining high-level posture descriptors, the methodology requires that the cat be standing and that the image depicts a side view. To this end, we employ a second lightweight CNN with the same architectural design as the overall posture classifier, but trained for binary classification to identify valid side-view representations of the cat. Like the posture classifier, this model operates on DensePose segmentation maps and is also described in Section 4. As a result, images where the cat is either not standing or not shown in a side view are excluded from further processing.

In the next step, the cropped RGB images are given as input to a pose estimation model known as MMPose (11). This model generates a skeleton for each image, providing the coordinates of the cat's body joints along with the confidence score for each estimation.

It is important to note that both the segmented images produced by DensePose and the joint positions estimated by MMPose may contain inaccuracies due to occlusions; variations in fur, texture, and color; unconventional postures; or limitations in the models' ability to generalize to feline anatomy. To address these issues, the subsequent step cross-validates and refines the detections by combining the two outputs, filtering out inconsistent or erroneous results. Although MMPose provides anatomically meaningful skeletal landmarks, the posture descriptors considered in this work are primarily assessed from the external profile of the animal in side view. In particular, back curvature and tail configuration depend on contour information that is not fully represented by the available skeletal keypoints. Furthermore, MMPose does not provide an explicit tail-tip landmark and does not directly encode the dorsal contour required for curvature estimation. For these reasons, DensePose-derived silhouettes constitute the primary geometric representation used for posture analysis, while MMPose is employed in a complementary role to validate anatomical consistency and improve the reliability of feature extraction.

This process yields a consistent set of geometric features, such as anatomical keypoints and silhouettes. Finally, a set of classifiers is applied to the validated posture data to infer high-level descriptors, as detailed in Section 6.

## 4 Overall posture and side view classifiers

**Figure 5:**
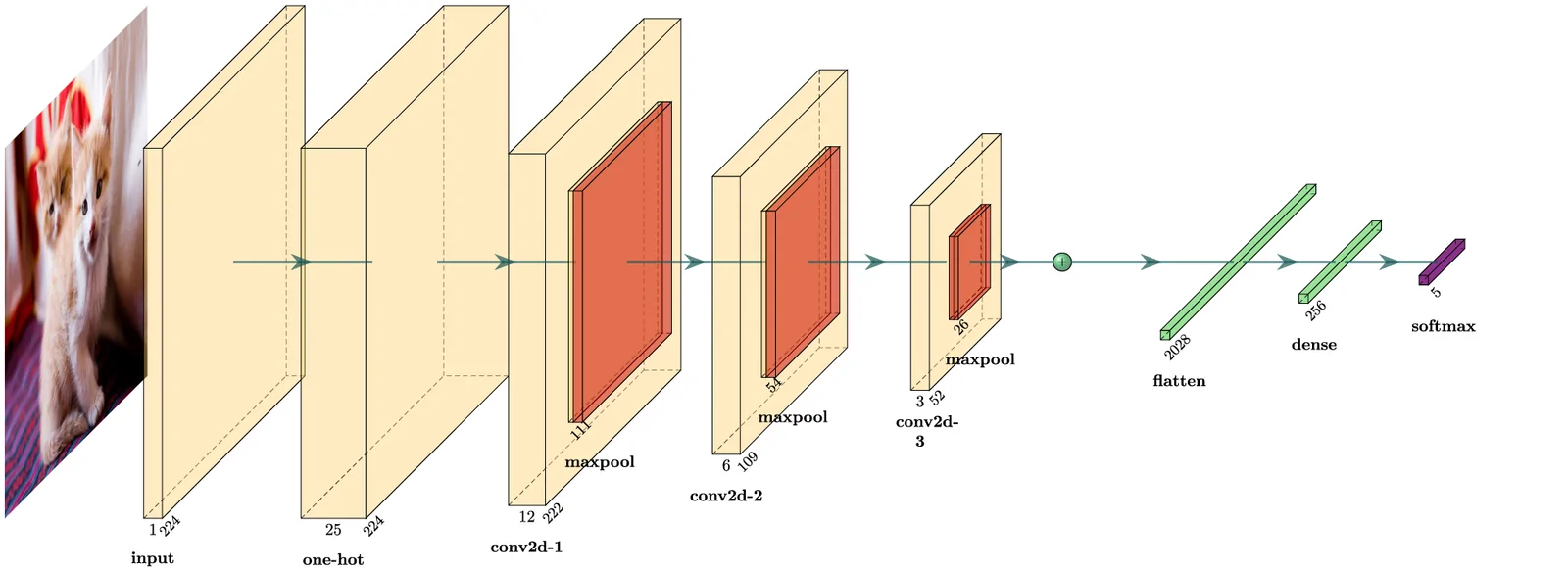
Architectures of the proposed lightweight CNNs for overall posture classification and side-view validation.

To classify the cat's overall posture and validate its side-view orientation prior to geometric analysis, we trained two lightweight Convolutional Neural Networks using supervised learning. Both networks take as input the segmentation maps produced by the DensePose model, resized to 224 × 224 pixels. Each pixel in these maps is assigned an integer in the range [0, 24], where 0 represents the background and the remaining values correspond to 24 distinct body parts of the cat. The detailed architecture is illustrated in Figure 5.

To enable effective application of convolutional filters, we introduce a custom layer that converts each segmentation map from a tensor of shape 224 × 224 × 1 into a one-hot encoded binary tensor of shape 224 × 224 × 25. This transformation prevents the network from treating anatomical labels as ordinal quantities and enables convolutional filters to learn spatial relationships across distinct anatomical regions.

The subsequent layers follow a conventional CNN structure: convolutional layers for feature extraction, pooling layers for dimensionality reduction, a flattening layer, a dense layer with 256 units and ReLU activation, and a final softmax layer that outputs the predicted class.

**Figure 6:**
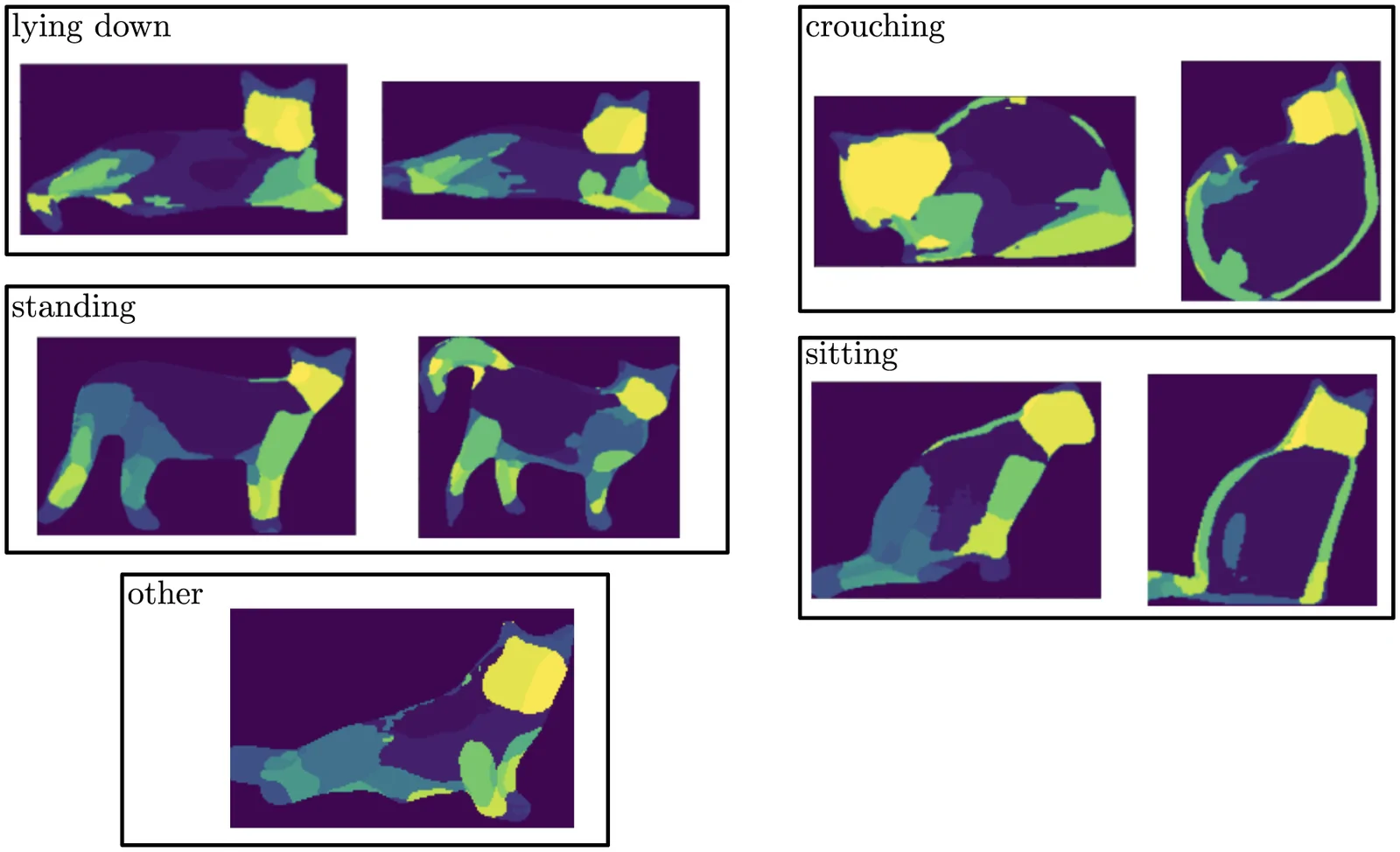
Examples of overall posture images from distinct classes, color-coded according to DensePose segments.

Specifically, the overall posture classifier takes segmentation maps as input and predicts one of five cat postures: lying down, crouching, standing, sitting, or other, as illustrated in Figure 6. This architecture was designed to maintain a low parameter count (approximately 524 thousand parameters), enabling efficient training and inference while maintaining competitive classification performance.

**Figure 7:**
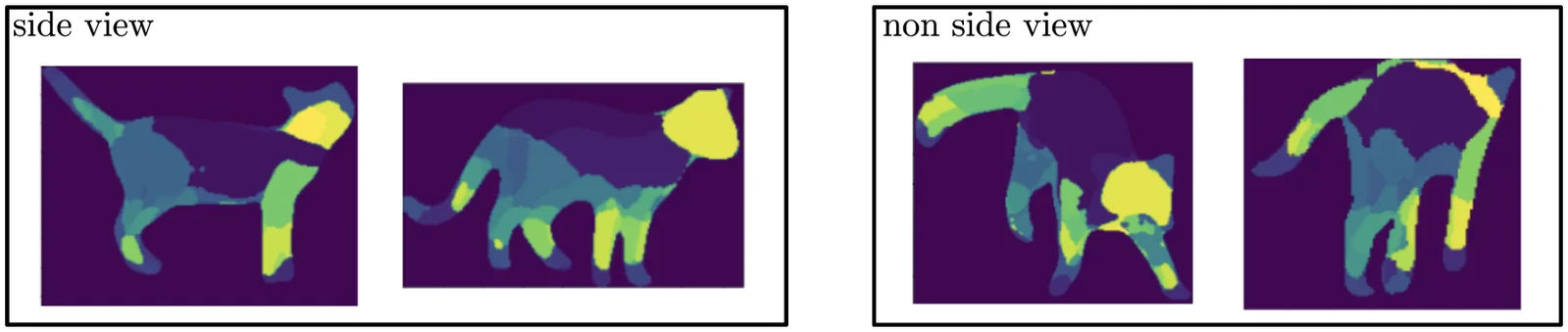
Examples of the side view and non side view images, color-coded according to DensePose segments.

The side-view classifier shares the same input representation but performs binary classification, determining whether the image corresponds to a valid side-view configuration. Examples of both classes are shown in Figure 7.

## 5 Geometric feature extraction for posture analysis

This section describes how geometric features are extracted from side-view images of cats, forming the foundation for computing high-level posture descriptors. To ensure consistency in posture estimation, outputs from DensePose and MMPose are cross-validated during this step. Frames in which the two models produce conflicting results are discarded.

### 5.1 Anatomical keypoints

**Figure 8:**
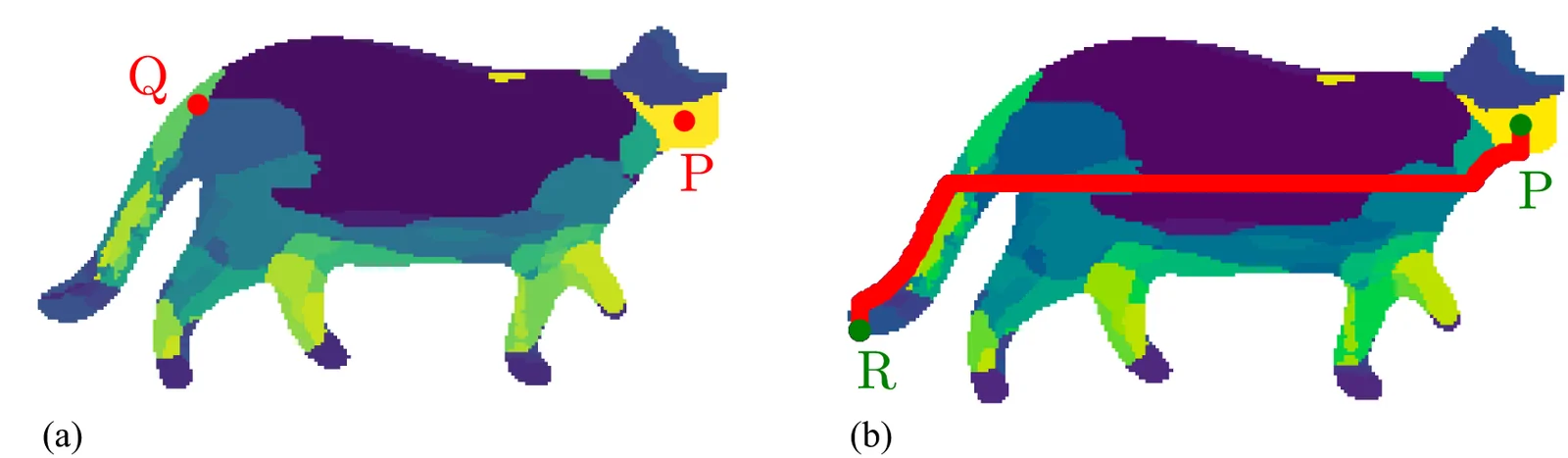
Illustration of anatomical reference points AND shortest-path analysis on the cat body mask. **(a)** Centroids of the head and base of the tail. **(b)** Shortest path from the head point P to point R, defined as the body pixel farthest from P in terms of unit-weighted shortest-path distance on the foreground pixel graph.

**Figure 9:**
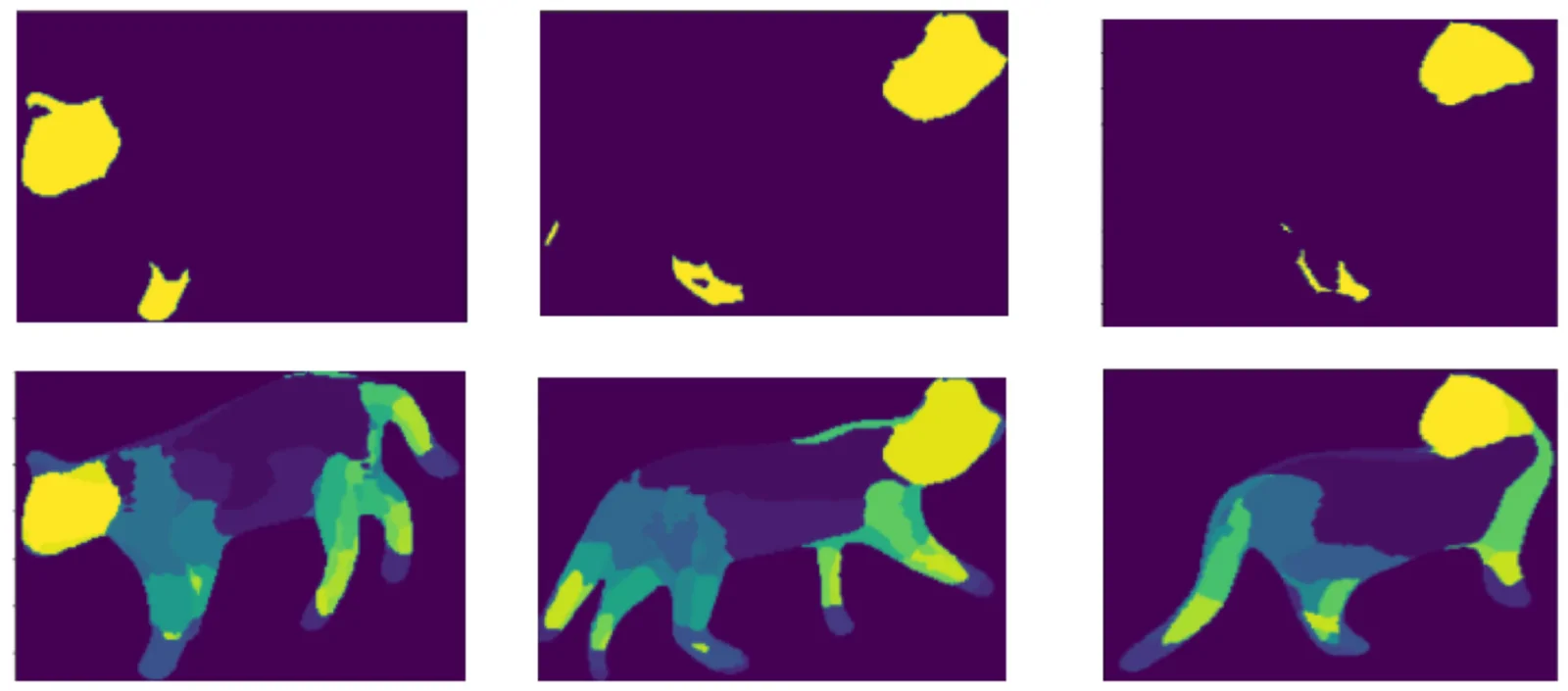
DensePose segments 6, 23, and 24 **(top)**, and all segments **(bottom)**.

The geometric feature extraction begins by identifying the centroid of the head (point *P*) and the base of the tail (point *Q*), as illustrated in Figure 8a. To determine point *P*, we cross-validate information from the DensePose segments and the MMPose skeleton. An empirical analysis of segmented images revealed that segments 6, 23, and 24 typically correspond to regions of the cat's head, although they occasionally represent other body parts, as shown in Figure 9. If the MMPose facial joints (eyes and nose) lie within these segments, the image is considered valid. The centroid of the largest among these DensePose segments is then selected as point *P*. To determine the base of the tail position (point *Q*), we directly use its corresponding joint provided by MMPose.

The next step is to identify the centroid of the tail tip (point *R*). Since MMPose does not include a joint corresponding to this anatomical landmark, we propose a custom algorithm that operates solely on the DensePose output. The algorithm assumes that the tail tip corresponds to the DensePose-identified body pixel that is farthest from the head point *P* in terms of shortest-path distance on the foreground pixel graph.

**Figure 10:**
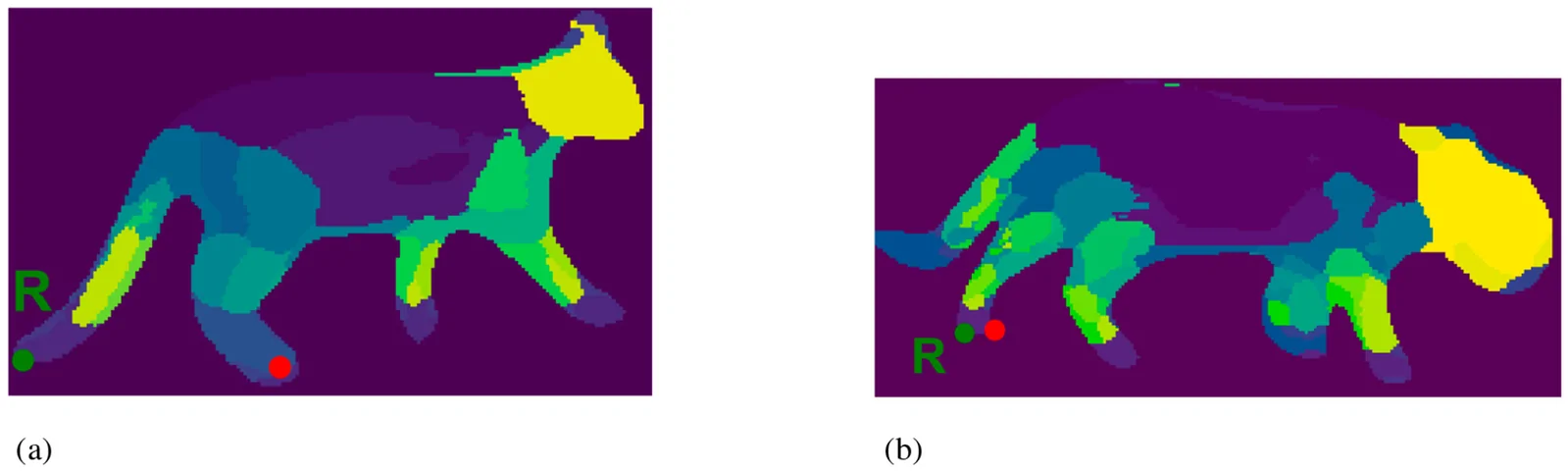
Examples of tail tip localization based on shortest-path distance from the head point on the foreground pixel graph. In successful cases, the identified point *R* corresponds to the anatomical tail tip and is spatially distant from the hind paw keypoints. **(a)** The point R (in green), identified as the pixel farthest from the head point based on unit-weighted shortest-path distance on the foreground pixel graph, correctly corresponds to the anatomical tail tip. It is also distant from the hind paw keypoint (in red). **(b)** The point R (in green), though far from the head, does not correspond to the tail tip. This misidentification is detected by measuring its proximity to the hind paw keypoint (in red).

We construct a graph where each node represents a pixel classified as part of the cat's body. Initially, all image pixels are treated as nodes and connected to their 4-connected neighbors, forming a graph that reflects the 2D spatial structure of the image. Background pixels are then removed, resulting in a graph composed exclusively of connected foreground (body) pixels. Dijkstra's algorithm is applied to this graph to compute unit-weighted shortest-path distances from *P*. The node with the greatest shortest-path distance is identified as point *R*. In most side-view images, this node corresponds to the tip of the tail (see Figure 8b). However, in some cases, due to occlusions or certain poses, the algorithm may incorrectly identify a hind paw. To detect such errors, we compare the location of *R* with the hind leg keypoints provided by the MMPose skeleton. If the Euclidean distance between *R* and any hind leg keypoint falls below a given threshold, we assume that the tail tip was misidentified. In these cases, the corresponding image is discarded from the dataset, as illustrated in Figure 10.

### 5.2 Silhouette extraction and segmentation

**Figure 11:**
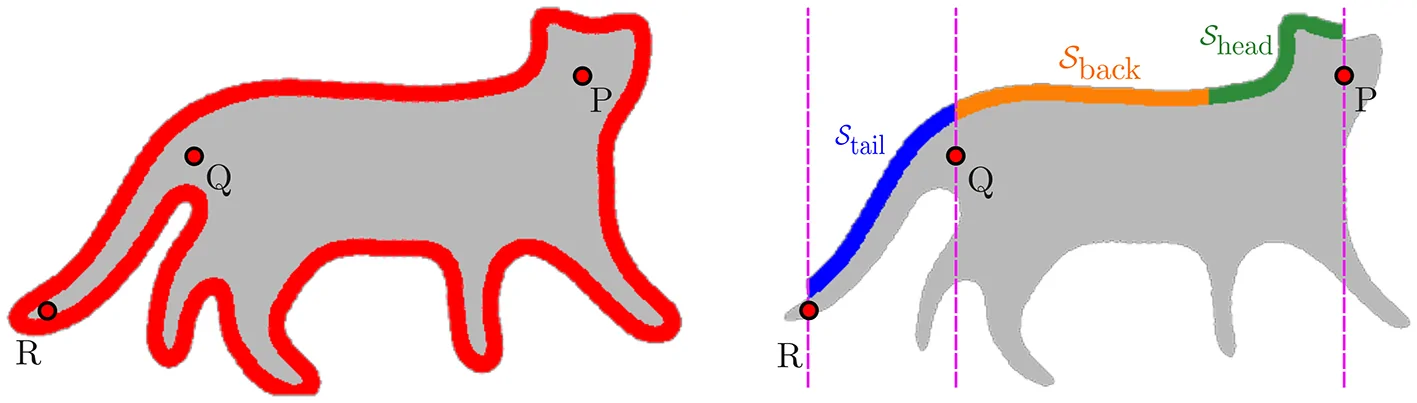
Silhouette extraction and segmentation: the silhouette of the cat is first extracted **(left)**, and then segments corresponding to the tail ($S_{\text{tail}}$), back ($S_{\text{back}}$), and head ($S_{\text{head}}$) are identified using vertical lines through points *P*, *Q*, and *R* **(right)**.

After estimating the keypoints *P*, *Q*, and *R*, we extract the silhouette of the cat by applying a contour detection algorithm to the union of DensePose segments, following the method of Suzuki and Abe (48). From this silhouette, we isolate three segments of the top boundary curve that correspond to the shape of the tail ($S_{\text{tail}}$), back ($S_{\text{back}}$), and head ($S_{\text{head}}$). The segment $S_{\text{tail}}$ traces the upper outline between the intersections of the silhouette with vertical lines passing through points *R* and *Q*. The portion of the curve between the vertical lines at *Q* and *P* is further divided into two parts: the first two-thirds define $S_{\text{back}}$, representing the back, while the remaining one-third nearest to point *P* defines $S_{\text{head}}$, capturing the head region. This segmentation procedure is illustrated in Figure 11.

## 6 High-level posture feature extraction and classification

In this stage, high-level posture features are derived from anatomical keypoints and silhouette segments. These geometric features are subsequently fed into rule-based classifiers to infer categorical postural states.

### 6.1 Head pitch classifier

**Figure 12:**
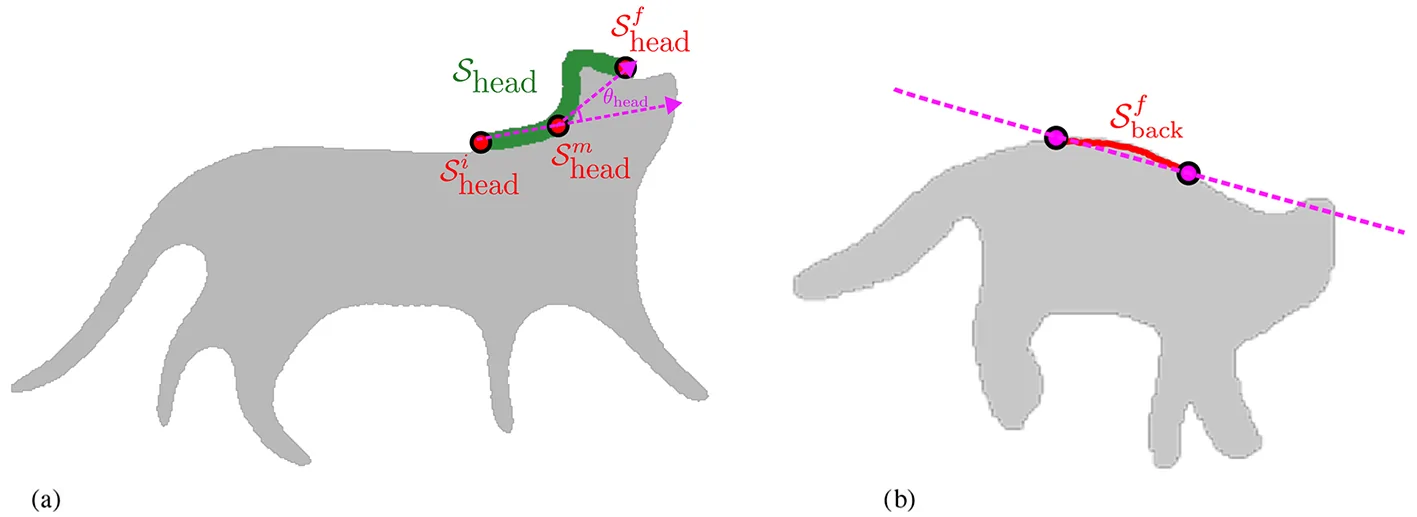
Extraction of posture features from silhouette segments: head pitch and back inclination are derived from $S_{\text{head}}$ and $S_{\text{back}}$, respectively. **(a)** Head pitch angle estimation using three keypoints from $S_{\text{head}}$. **(b)** Back inclination estimation using $S_{\text{back}}$.

To quantify head pitch, we define the head segment $S_{\text{head}}$ based on three anatomically meaningful points extracted from the silhouette: the initial ($S_{\text{head}}^{i}$), middle ($S_{\text{head}}^{m}$), and final ($S_{\text{head}}^{f}$) positions along the head contour, as shown in Figure 12a. The head pitch angle, θ_head_, is defined as the signed angle between the vectors $\vec{S_{\text{head}}^{i}S_{\text{head}}^{m}}$ and $\vec{S_{\text{head}}^{m}S_{\text{head}}^{f}}$, computed using the 2D cross product to determine its sign. This signed formulation preserves the direction of rotation about the lateral axis of the head in the sagittal plane, allowing positive and negative pitch values to be distinguished.

**Figure 13:**
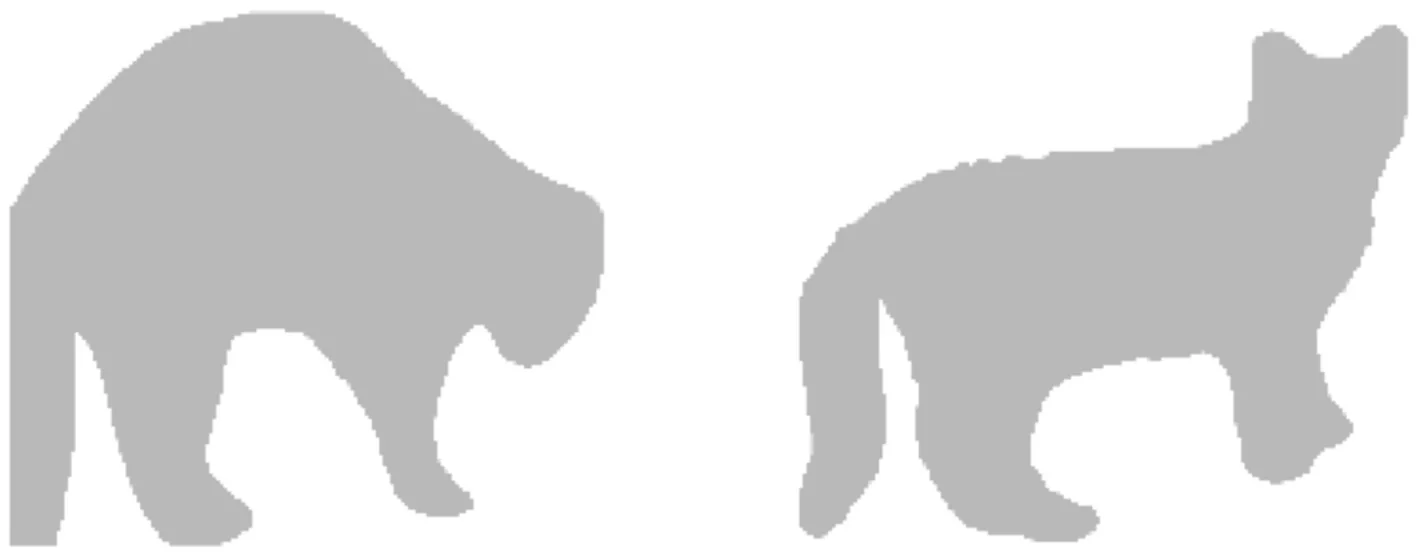
Examples of each class of head pitch: down **(left)** and up **(right)**.

To categorize head orientation into one of the semantic classes *down* or *up*, as illustrated in Figure 13, we employ a rule-based classifier *f*_head_. This classifier discretizes the continuous pitch angle θ_head_ using an empirically learned threshold, *T*_head_. The threshold is estimated from a labeled dataset:

$$\begin{array}{l} D_{\text{head}}={\left\{\left(\theta_{i},h_{i}\right)\right\}}_{i=1}^{N}, \end{array}$$

where θ_*i*_ is a sample head pitch angle and *h*_*i*_∈{down, up} is its corresponding class. The estimation is based on a cross-validation procedure aimed at maximizing inter-class separation and classification performance on held-out data.

The classifier *f*_head_ is defined as:

$$f_{\text{head}}\left(\theta \right)=\{\begin{array}{cc} \text{down}, & \text{if }\theta <T_{\text{head}}\\ \text{up},\; & \text{if }\theta \geq T_{\text{head}} \end{array}$$

### 6.2 Back classifiers

Two posture-related features are extracted from the back: the inclination *m*_back_ and the curvature κ_back_. Both are derived from $S_{\text{back}}$, which approximates the back's shape.

The back inclination *m*_back_ is estimated by leveraging the side-view constraint (Section 4). It is computed as the slope of the line connecting the endpoints of $S_{\text{back}}$, relative to the horizontal axis of the image, as shown in Figure 12b.

**Figure 14:**
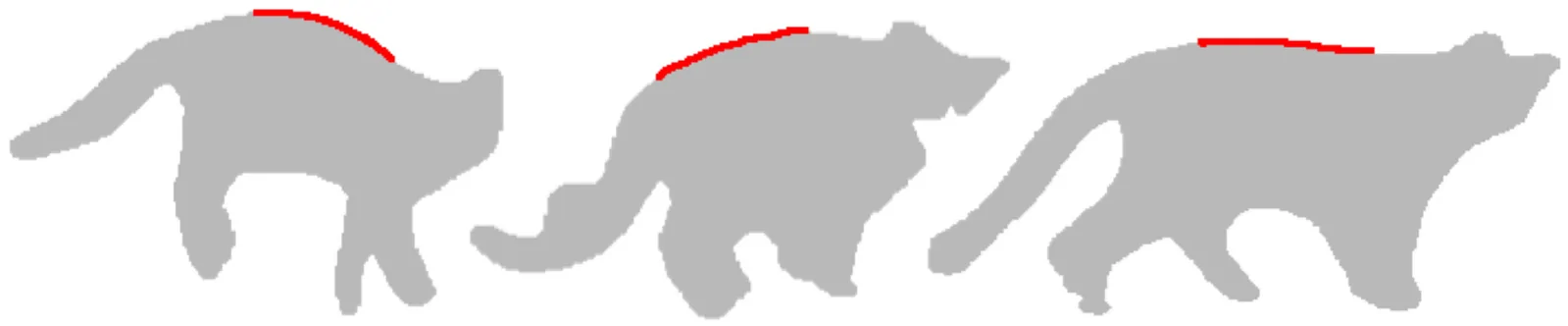
Examples of each class of back inclination: negative **(left)**, positive **(middle)** and neutral **(right)**.

Similar to the head pitch classifier, we define a back inclination classifier *f*_back_, which uses two thresholds $T_{\text{back}}^{d}$ and $T_{\text{back}}^{u}$ learned from a labeled training set. This classifier maps the inclination *m*_back_ to one of three posture classes: *negative, neutral*, or *positive*, as illustrated in Figure 14. Formally, it is defined as

$$f_{\text{back}}\left(m\right)=\{\begin{array}{cc} \text{negative}, & \text{if }m<T_{\text{back}}^{d}\;\\ \text{neutral},\; & \text{if }T_{\text{back}}^{d}\leq m\leq T_{\text{back}}^{u}\\ \text{positive}, & \text{if }m>T_{\text{back}}^{u}.\; \end{array}$$

**Figure 15:**
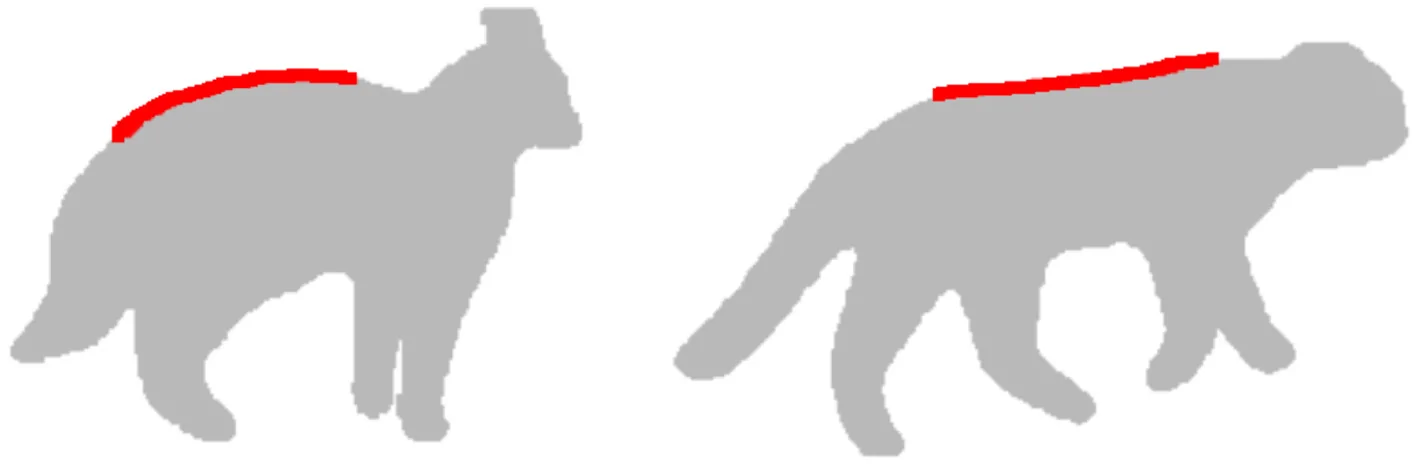
Illustrative examples of back curvature: a cat with pronounced arched back **(left)** and a cat with non-arched back **(right)**.

Back curvature, κ_back_, is estimated by applying Principal Component Analysis (PCA) (49) to the set of points in the discrete curve $S_{\text{back}}$, represented as a matrix with each row corresponding to a 2D point. PCA identifies the directions of maximum variance: the first principal component captures the back's main orientation, while the second reflects orthogonal deviations. The eigenvalue associated with the second component serves as an indirect measure of curvature: small values indicate a nearly straight segment, while larger values suggest increased bending. Figure 15 shows two distinct examples of back curvature.

The back curvature classifier *g*_back_ performs binary classification based on a single threshold *T*_curv_, which is learned from supervised data. It evaluates the curvature κ_back_ and classifies the back as either *arched* or *non-arched*. Formally,


$$g_{\text{back}}\left(\kappa \right)=\{\begin{array}{cc} \text{arched},\; & \text{if }\kappa >T_{\text{curv}}\\ \text{non-arched}, & \text{otherwise}. \end{array}$$

### 6.3 Tail classifier

**Figure 16:**
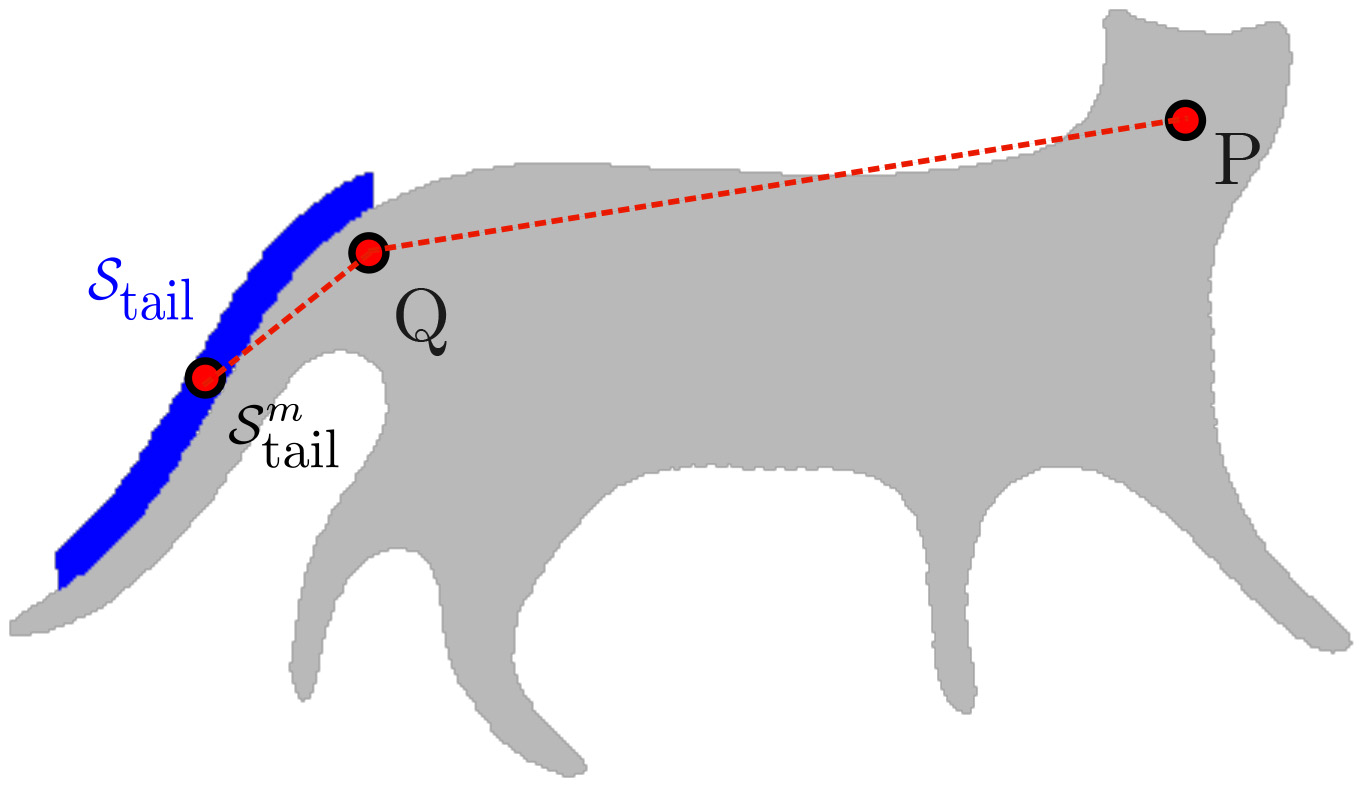
Tail inclination estimation using anatomical keypoints *P*, *Q* and $S_{\text{tail}}^{m}$.

Finally, we calculate the tail angle θ_tail_ based on three reference points: the head point *P*, the tail base point *Q*, and the midpoint $S_{\text{tail}}^{m}$ of the tail curve $S_{\text{tail}}$. We use the midpoint $S_{\text{tail}}^{m}$ instead of the tail endpoint to mitigate errors caused by complex tail configurations. For example, a tail that initially curves upward might later bend downward, leading to an incorrect horizontal or downward classification if only the endpoint were considered. The tail angle θ_tail_ is then estimated as the angle between vectors $\vec{S_{\text{tail}}^{m}Q}$ and $\vec{QP}$, as illustrated in Figure 16.

**Figure 17:**
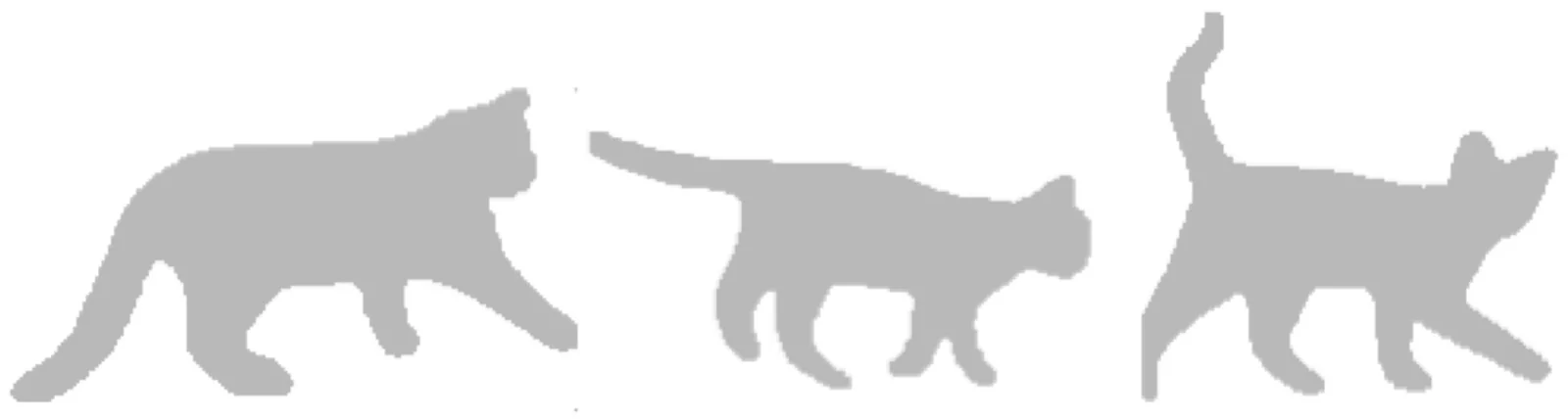
Examples of tail configurations classified as down **(left)**, neutral **(middle)**, and up **(right)**.

The tail configuration classifier *f*_tail_ operates on the tail angle θ_tail_ to predict one of three semantic classes: *down, neutral*, or *up* (see Figure 17). It requires two thresholds ($T_{\text{tail}}^{d}$ and $T_{\text{tail}}^{u}$), which are learned from a supervised training set. Formally, *f*_tail_ is defined as:

$$f_{\text{tail}}\left(\theta \right)=\{\begin{array}{cc} \text{down}, & \text{if }\theta <T_{\text{tail}}^{d}\;\\ \text{neutral}, & \text{if }T_{\text{tail}}^{d}\leq \theta \leq T_{\text{tail}}^{u}.\\ \text{up},\; & \text{if }\theta >T_{\text{tail}}^{u}\; \end{array}$$

## 7 Experimental evaluation

In this section, we present experiments designed to address the following research questions:

RQ1. How accurately can the proposed lightweight neural networks detect side views and overall postures of cats from DensePose-based segmentation maps, and how does their performance compare to larger VGG19-based architectures under the same staged-processing conditions?
RQ2. What is the impact of MMPose-assisted anatomical consistency validation on the proportion of frames eligible for geometric posture analysis?
RQ3. How accurately can the proposed classifiers predict high-level postures?
RQ4. In real-world video streams, what proportion of frames can be expected to successfully pass all detection, validation, and filtering stages of the pipeline, under representative operating conditions?
RQ5. How accurately does the end-to-end computer vision pipeline estimate and classify feline posture descriptors from real-world video sequences, considering only frames that successfully pass all detection, validation, and filtering stages?
RQ6. How can the automatically extracted posture descriptors be used to characterize and compare behavioral trajectories in real-world observations?

The following subsections address each question in detail, presenting the experimental setup, evaluation metrics, and results to systematically validate the performance of the proposed pipeline.

### 7.1 Evaluating side-views and overall postures classifiers

To address **RQ1**, we evaluated the classification performance of the proposed lightweight CNNs and compared them against substantially larger pretrained architectures based on a VGG19 backbone. The objective of this comparison is to determine whether the proposed compact task-specific architectures can achieve competitive or superior performance relative to deeper, high-capacity networks.

For each task (side-view and overall posture classification), two models were trained and evaluated under identical data splits and training protocols: (i) the proposed lightweight CNN architecture described in Section 4; and (ii) a VGG19-based architecture composed of a pretrained VGG19 convolutional backbone (initialized with ImageNet weights), followed by the same classification head used in the proposed lightweight CNN (i.e., a dense layer with 256 ReLU-activated units and a final output layer with softmax activation for multi-class classification or sigmoid activation for binary classification).

Each task was associated with a distinct RGB image dataset collected using low-cost cameras operating at a minimum resolution of 1280 × 720 pixels. Although the datasets differ in target classes and labeling criteria, they were acquired under similar environmental conditions. The class distributions for both datasets are summarized in Tables 1, 2.

**Table 1:** Class distribution for the side-view classification dataset.

Class	Train	Test
Side-view	332	333
Not side-view	86	86
Total	418	419

**Table 2:** Class distribution for the overall posture classification dataset.

Class	Train	Test
Lying down	40	39
Sitting	340	341
Crouching	48	49
Standing	92	93
Other	14	13
Total	534	535

In the VGG19-based models, the convolutional backbone was fine-tuned during training. This architecture contains approximately 20 million trainable parameters, representing a substantially higher model capacity compared to the approximately 0.52 million parameters of the proposed lightweight networks.

All models were trained using the Keras framework with the Adam optimizer and categorical cross-entropy loss (binary cross-entropy for side-view detection). Early stopping based on validation loss was applied to mitigate overfitting. To address class imbalance, class weighting was applied during training by assigning weights inversely proportional to the class frequencies. Model performance was quantitatively assessed using accuracy, F1-score, and the confusion matrix, which together provided complementary insights into both overall and class-wise predictive performance.

As shown in Tables 3, 4, the proposed lightweight CNNs achieved higher classification accuracy than the substantially larger VGG19-based architectures in both tasks. Notably, the lightweight models contain approximately 38 times fewer parameters while attaining superior predictive performance.

**Table 3:** Comparison between the VGG19-based model and the proposed lightweight CNN for side-view classification.

Model	Accuracy	F1-score	# Parameters
VGG19	91.9%	91.4%	20,156,226
Proposed CNN	92.6%	92%	523,469

**Table 4:** Comparison between the VGG19-based model and the proposed lightweight CNN for overall posture classification.

Model	Accuracy	F1-score	# Parameters
VGG19	91.0%	90.4%	20,156,997
Proposed CNN	93.1%	91.8%	524,240

These findings indicate that increasing model depth and parameter count does not necessarily improve performance when operating on structured segmentation maps derived from DensePose. Instead, compact architectures specifically designed for anatomical segmentation inputs appear to generalize more effectively for the considered posture classification tasks.

**Figure 18:**
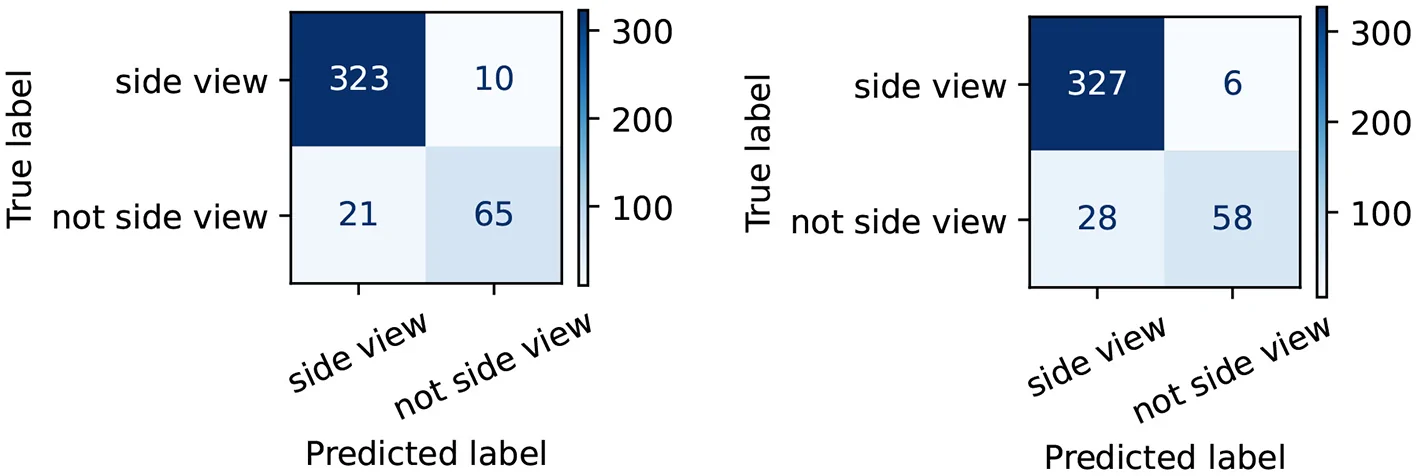
Confusion matrices for the binary side-view classification task, comparing the proposed lightweight CNN **(left)** with the VGG19-based baseline **(right)**.

**Figure 19:**
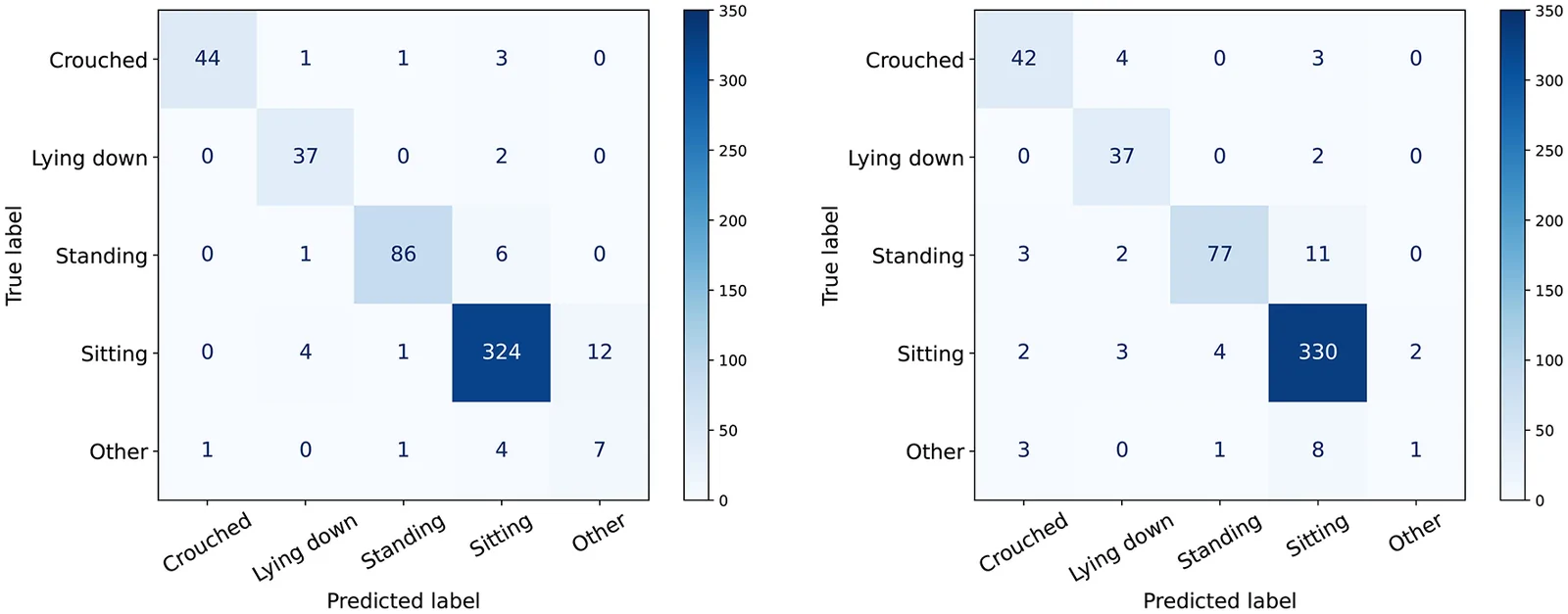
Confusion matrices for the five-class overall posture classification task, comparing the proposed lightweight CNN **(left)** with the VGG19-based architecture **(right)**.

To provide a more detailed analysis of classification behavior, Figures 18, 19 present the confusion matrices for both the lightweight and VGG19-based architectures in the side-view and overall posture tasks, respectively.

The confusion matrices further illustrate the comparative behavior of the models. For side-view classification, the lightweight CNN correctly classified 323 side-view and 65 not-side-view images, while the VGG19-based model correctly classified 327 side-view but only 58 not-side-view samples, indicating a higher misclassification rate for the minority class.

For overall posture classification, the lightweight CNN achieved stronger diagonal dominance across classes, correctly identifying 86 standing and 7 other samples, compared to 77 and 1, respectively, for the VGG19-based model. The VGG19 architecture also exhibited increased confusion between visually similar postures, particularly standing and sitting (11 standing samples misclassified as sitting). Overall, the lightweight models demonstrate more balanced class-wise performance in addition to higher accuracy.

In summary, these findings provide a positive answer to **RQ1**. The proposed lightweight CNNs achieved performance comparable to, and in these experiments slightly higher than, VGG19-based architectures while using approximately 38 times fewer trainable parameters. This result should be interpreted in the context of the staged design of the proposed framework. The preceding detection, cropping, and DensePose segmentation steps progressively simplify the classification problem by converting raw RGB images into structured anatomical representations. Consequently, the later classification stages do not require the capacity of a large general-purpose visual backbone. Instead, compact task-specific architectures are sufficient to learn the relevant spatial relationships in the segmentation maps, making them more suitable for efficient deployment within the proposed pipeline.

It should be noted that the VGG19-based architecture was selected as a deliberately high-capacity, general-purpose baseline, in order to directly test the hypothesis that compact, task-specific networks operating on DensePose-derived segmentation maps can match or exceed the performance of substantially larger, generically pretrained backbones. The comparison was therefore not intended as an exhaustive benchmark against the current state of the art in image classification; a broader comparison encompassing additional recent lightweight and efficient architectures (e.g., MobileNet- or EfficientNet-family models) is identified as a direction for future work.

### 7.2 Evaluating high-level posture classifiers

In this subsection, we investigate **RQ2** and **RQ3**. For this purpose, we consider an initial dataset of 474 side-view images of standing cats, used for training and evaluating the high-level posture classifiers (Section 6). It is important to note that all images in this dataset had already successfully passed the object detection, DensePose segmentation, and side-view validation stages of the pipeline.

This initial dataset was then subjected to anatomical consistency validation during the geometric feature extraction step (Section 5). Specifically, images were discarded when inconsistencies were observed between the anatomical keypoints extracted by MMPose and the body-part segmentation provided by DensePose.

Table 5 summarizes the distribution of images before and after this MMPose-assisted validation process for each posture category, along with the corresponding retention percentages. Since all frames had already passed detection and side-view classification, the reduction in sample size reflects exclusively the selectivity introduced by anatomical consistency verification.

**Table 5:** Sample counts before and after anatomical consistency validation for each posture feature, showing retention rates after excluding frames with inconsistencies between MMPose keypoints and DensePose segmentation.

Posture	Pre-filtering	Post-filtering	Percentage (%)
Head pitch			
Head up	213	193	90.52
Head down	261	248	95.00
Back inclination			
Positive slope	97	95	97.94
Neutral slope	94	85	90.43
Negative slope	283	261	92.30
Back curvature			
Arched back	57	48	84.21
Non-arched back	417	393	94.24
Tail			
Tail down	417	414	99.4
Horizontal tail	13	13	100.00
Vertical tail	44	14	31.82

The results show that for most posture features, more than 90% of frames satisfy the anatomical validation criteria, indicating that the majority of side-view standing images support geometrically coherent descriptor extraction. However, specific configurations, most notably vertical tail positions (31.82% retention), exhibit substantially lower retention rates, revealing a higher sensitivity to precise anatomical alignment.

In response to **RQ2**, these findings demonstrate that MMPose-assisted validation constitutes a structural reliability mechanism within the proposed pipeline. By enforcing consistency between skeletal keypoints and DensePose segmentation, the validation stage ensures that geometric posture descriptors are computed only when anatomically coherent conditions are met. This mechanism selectively constrains configurations that are prone to skeletal-segmentation misalignment while preserving the majority of stable cases, thereby safeguarding the integrity of the geometric posture analysis.

It is important to emphasize that MMPose and DensePose play complementary roles in the proposed framework. While MMPose provides skeletal landmarks for anatomical consistency verification, DensePose is indispensable for detailed body-part segmentation, particularly for tail-region identification. Since MMPose does not include explicit tail joints, removing DensePose would prevent the computation of tail-related geometric descriptors, making such a configuration infeasible.

We next evaluate the performance of high-level posture classifiers trained on the filtered dataset to estimate the proposed posture features, including head pitch, back inclination and curvature, and tail position.

#### 7.2.1 Head pitch classifier analysis

**Figure 20:**
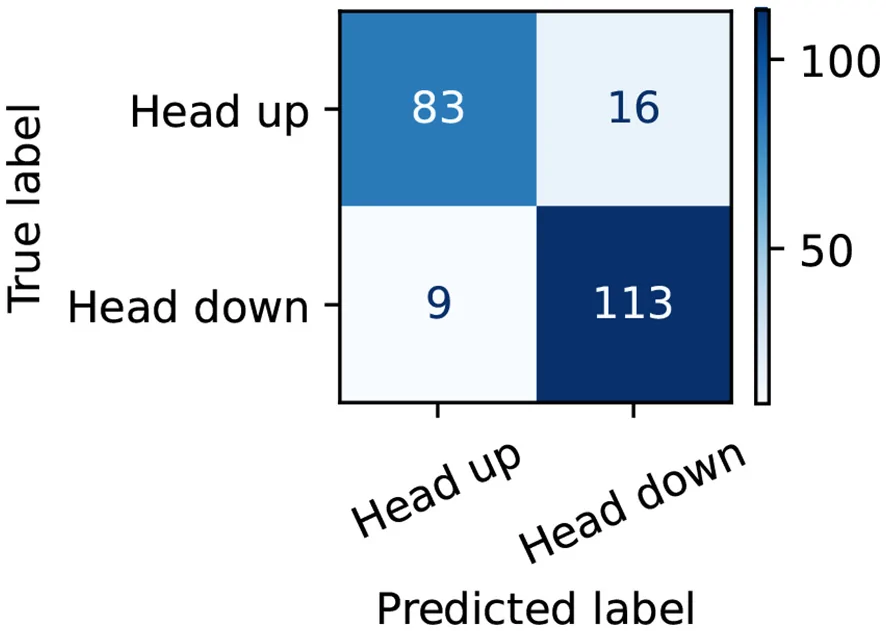
Confusion matrix for the binary head pitch classifier (upward vs. downward) on the test set.

Following the calibration procedure described in Section 6.1, the head pitch classifier was tuned to a threshold of *T*_head_ = −0.28 using 221 training images (50% of the initial dataset). The resulting confusion matrix (Figure 20) shows that the model correctly classified 196 out of 221 samples, yielding an overall accuracy of 88%. Specifically, 83 head-up and 113 head-down samples were correctly identified. Among the 25 misclassifications, 16 head-up samples were incorrectly predicted as head-down, while only 9 head-down samples were misclassified as head-up, indicating a slight bias toward the majority class.

This behavior is reflected in the per-class F1-scores reported in Table 6, with values of 0.87 for the head-up class and 0.90 for the head-down class. Despite the class imbalance, both classes achieved high F1-scores, demonstrating that the classifier maintains a good balance between precision and recall. Overall, these results confirm that the calibrated threshold provides a robust and reliable separation between upward and downward head poses. Future improvements could focus on balancing the dataset or applying data augmentation techniques to further enhance performance, particularly for the underrepresented head-up class.

**Table 6:** Per-class F1-scores for the binary head pitch classifier (head up vs. head down).

Class	F1-score
Head up	0.87
Head down	0.90

#### 7.2.2 Back classifiers analysis

**Figure 21:**
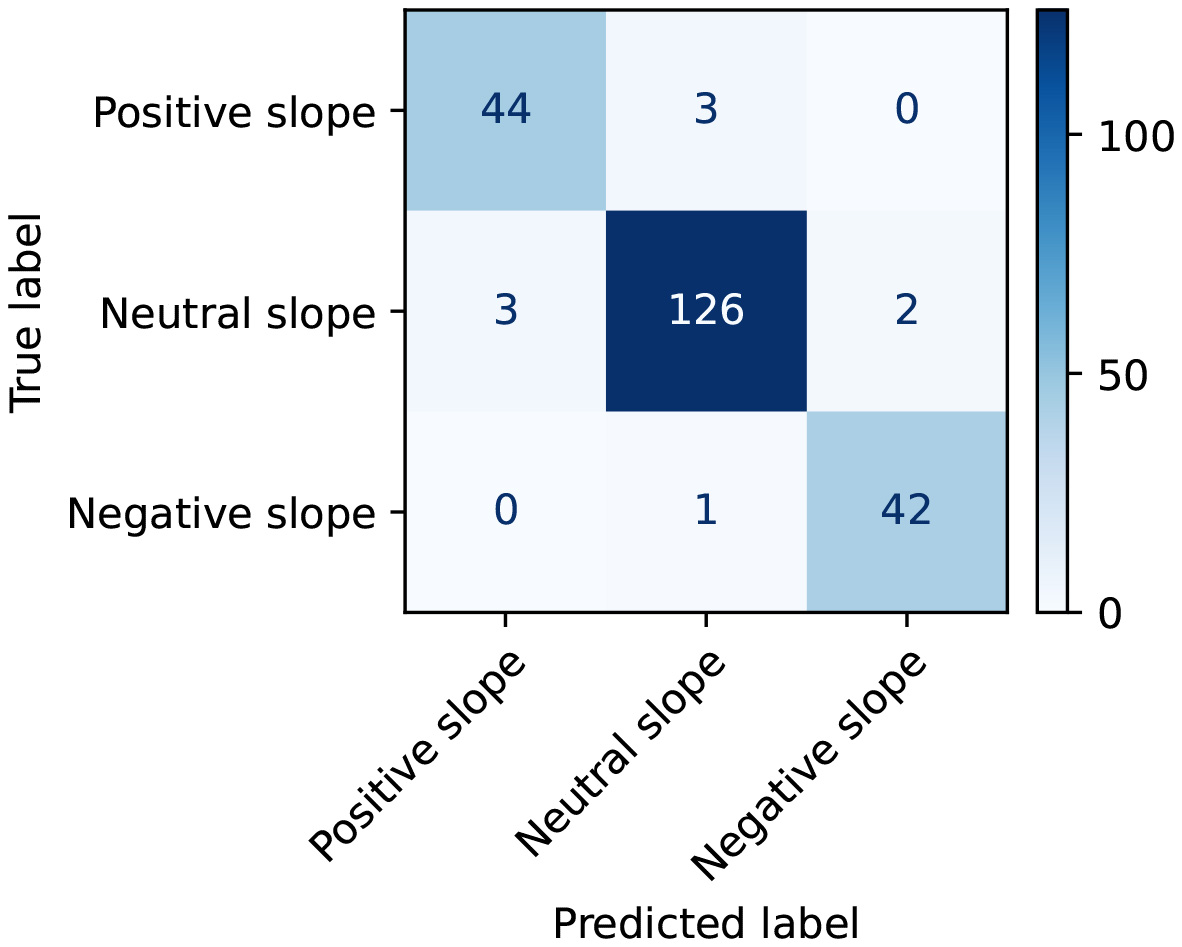
Confusion matrix for the back inclination classifier on the test set.

To classify back inclination, the decision thresholds were tuned through cross-validation to $T_{\text{back}}^{d}=-0.16$ and $T_{\text{back}}^{u}=0.15$. Using these values, the back inclination classifier achieved an overall accuracy of 95%, indicating strong global performance. A more detailed evaluation is provided through the confusion matrix shown in Figure 21 and the class-wise F1-scores reported in Table 7. The confusion matrix demonstrates that most samples are correctly classified, with only a small number of misclassifications occurring primarily between neighboring classes (e.g., *Positive slope* and *Neutral slope*), which is expected given the continuous nature of back inclination.

**Table 7:** Per-class F1-scores for the back inclination classifier.

Class	F1-score
Positive slope	0.94
Negative slope	0.97
Neutral slope	0.97

Consistently high F1-scores are observed across all classes, with *Negative slope* and *Neutral slope* achieving the highest values (0.97), while *Positive slope* presents a slightly lower yet still robust F1-score of 0.94. These results demonstrate balanced precision and recall for all classes and confirm that the selected thresholds enable reliable discrimination among back inclination states. Overall, the combination of high accuracy, strong diagonal dominance in the confusion matrix, and uniformly high F1-scores indicates that the classifier is well suited for real-time monitoring applications.

**Figure 22:**
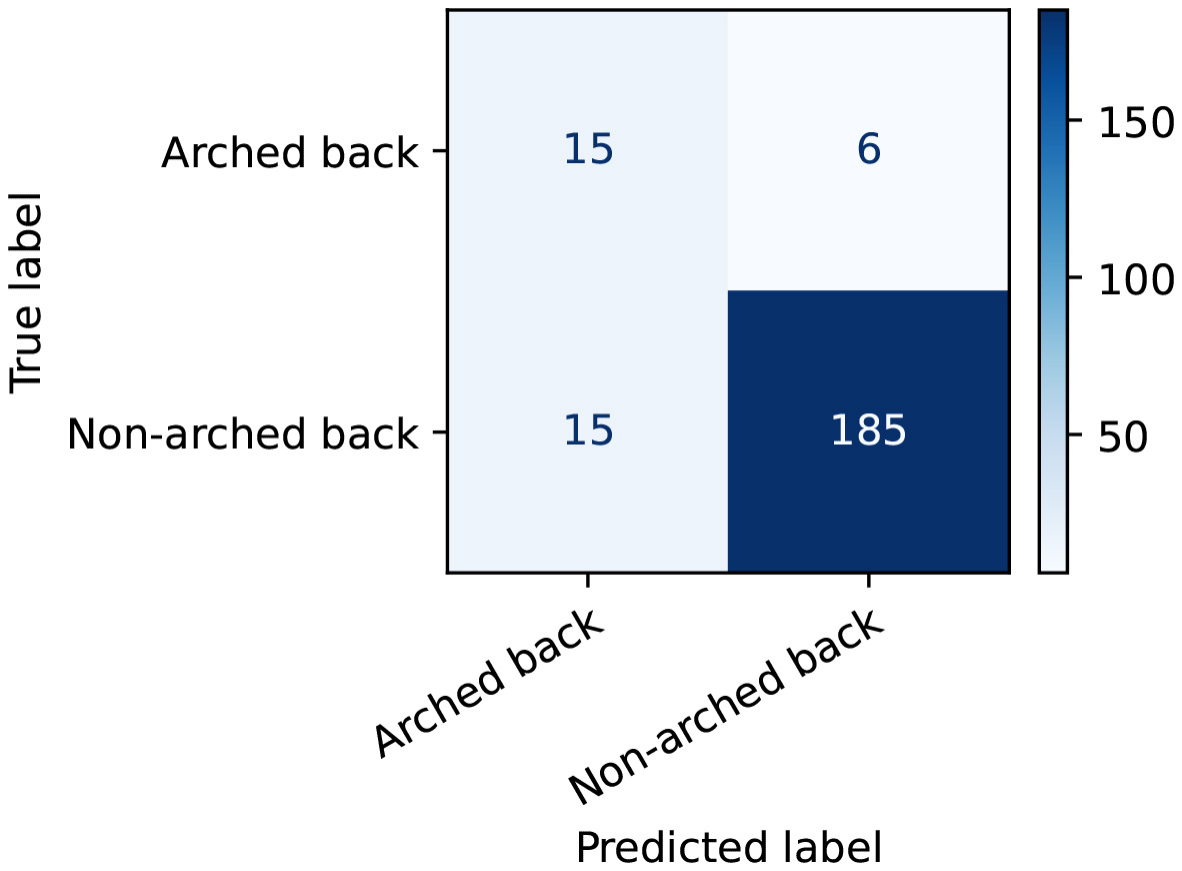
Confusion matrix for the back curvature classifier on the test set.

**Figure 23:**
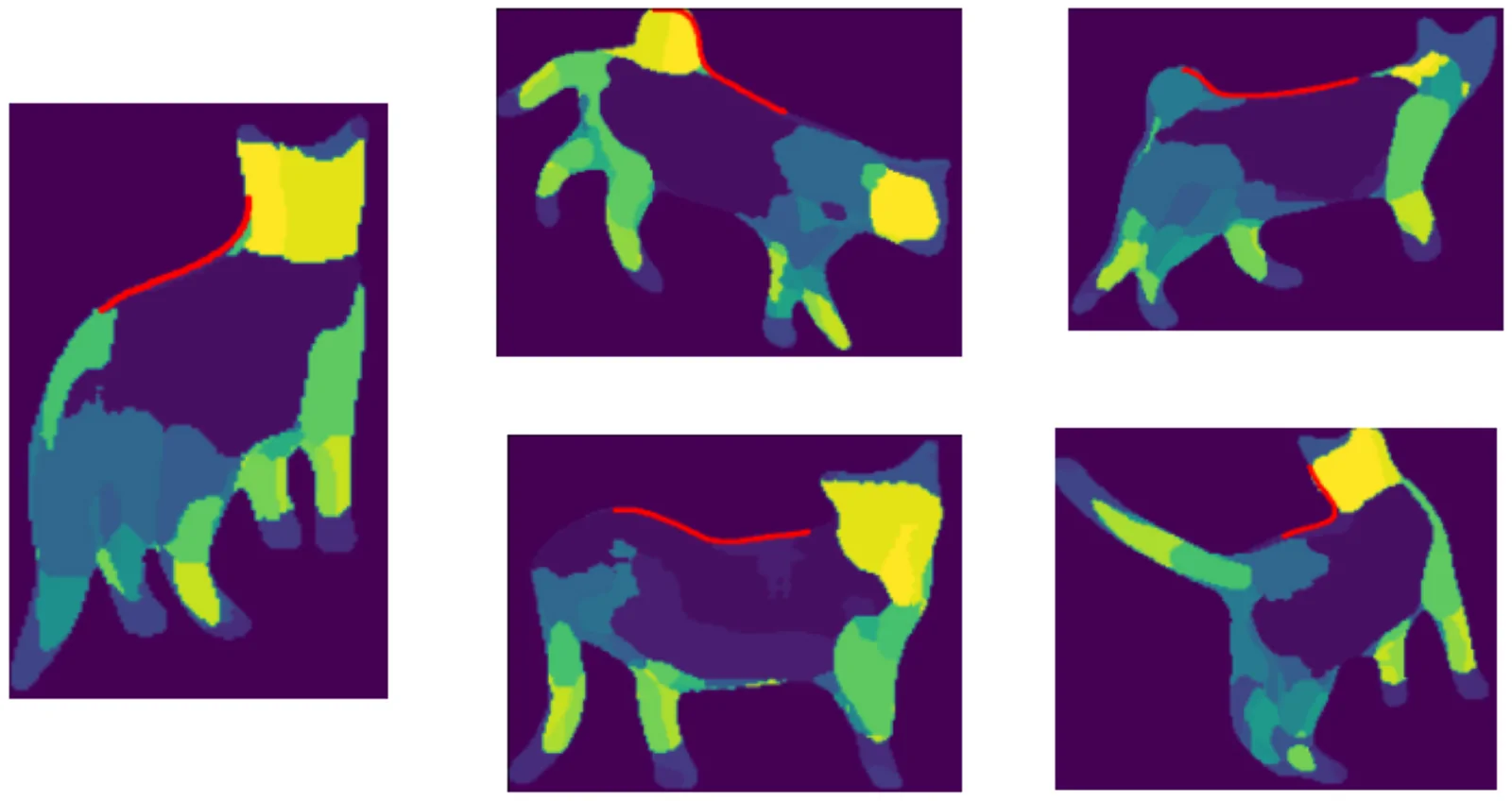
Examples of misclassified images in the curvature classification task. Misclassifications are primarily due to the cat's posture, which caused the curvature to be concentrated either toward the rear or forward regions (e.g., including the head), leading to inflated curvature values.

Regarding back curvature, a threshold of *T*_curv_ = 0.0095 was estimated through cross-validation on the training set to discriminate between arched and non-arched postures. Using this threshold, the curvature classifier achieved an overall accuracy of 90%. Nevertheless, the confusion matrix shown in Figure 22 highlights a pronounced class imbalance, with a substantially larger number of non-arched samples compared to arched ones. This imbalance leads to asymmetric classification performance: while non-arched backs are detected with high reliability, a significant portion of arched backs is misclassified as non-arched. This behavior is reflected in the per-class F1-scores reported in Table 8, where the arched back class achieves a considerably lower F1-score (0.59) than the non-arched class (0.95). Moreover, in some instances, localized abnormal segment curvature results in inflated curvature estimates, further contributing to back misclassification (Figure 23). Finally, these results suggest that augmenting the dataset to mitigate class imbalance, particularly by increasing the representation of arched back samples, could lead to more balanced performance and improved classification of arched postures.

**Table 8:** Per-class F1-scores for the back curvature classifier.

Class	F1-score
Arched back	0.59
Non-arched back	0.95

#### 7.2.3 Tail classifier analysis

**Figure 24:**
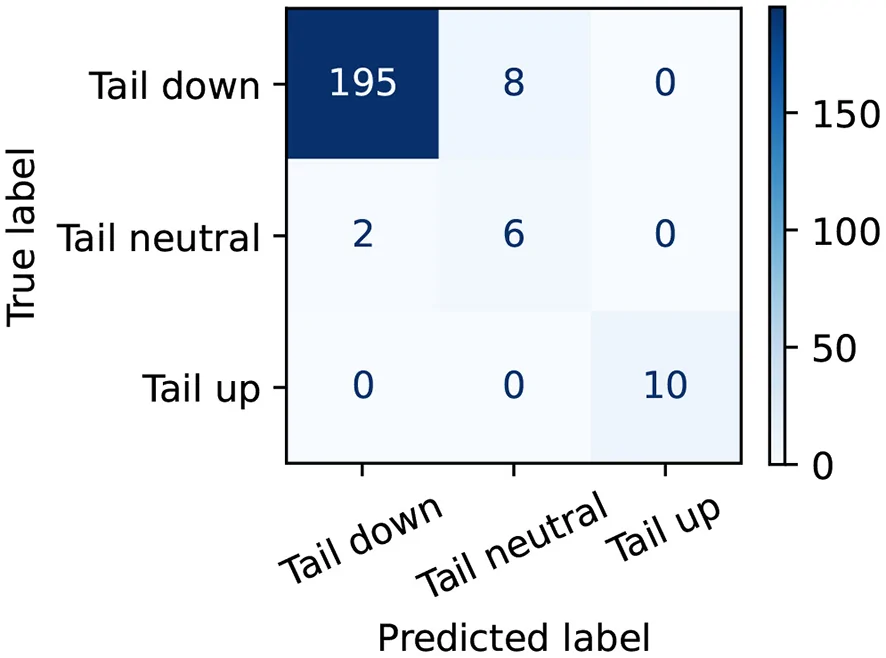
Confusion matrix for the tail configuration classifier on the test set.

For the tail configuration classifier *f*_tail_, the optimal decision thresholds were estimated as $T_{\text{tail}}^{d}=-0.33$ and $T_{\text{tail}}^{u}=0.21$. As shown in Figure 24, the classifier achieved an overall accuracy of 95.47% on the test set. The confusion matrix indicates perfect classification performance for the upward tail configuration, with all samples correctly identified. The downward tail class also exhibited very high performance, achieving a classification accuracy of approximately 96%.

In contrast, the neutral tail configuration attained a lower accuracy of 75%, with misclassifications occurring primarily between the neutral and downward classes. This reduced performance can be largely attributed to the strong class imbalance present in the training dataset, as well as the inherently subtle visual differences between neutral and slightly inclined tail positions. Such subtlety may have led to ambiguity in class boundaries and, consequently, to inconsistent annotations during dataset labeling. These factors jointly increase the difficulty of reliably distinguishing the neutral class. These trends are further corroborated by the per-class F1-scores reported in Table 9, where the tail-up class achieved a perfect F1-score of 1.00, the tail-down class reached 0.97, and the neutral class presented a notably lower F1-score of 0.55.

**Table 9:** Per-class F1-scores for the tail classifier.

Class	F1-score
Tail down	0.97
Neutral tail	0.55
Tail up	1.00

Overall, the tail configuration classifier demonstrated robust performance, particularly for clearly defined upward and downward tail orientations. While the neutral class remains more challenging due to class imbalance, subtle geometric variations, and potential annotation inconsistencies, the model consistently distinguished the primary tail configurations in the majority of cases. These results validate the effectiveness of the selected thresholds and support the classifier's suitability for real-time posture analysis, with further improvements possible through data rebalancing.

#### 7.2.4 Discussion

These results indicate that the proposed geometric and rule-based classifiers achieve strong overall performance across most posture components. Head pitch and back inclination were estimated with high reliability, achieving accuracies of 88% and 95%, respectively, with balanced F1-scores across classes. These results suggest that silhouette-based geometric features are well suited for capturing global postural trends in side-view images. Tail configuration was also classified accurately, particularly for clearly defined upward and downward orientations, which achieved near-perfect performance. In contrast, the neutral tail class proved more challenging, mainly due to class imbalance and the subtle visual differences between adjacent tail orientations.

Back curvature estimation achieved good overall accuracy but showed reduced performance for the curved class, reflecting both the limited number of curved samples and the sensitivity of the curvature measure to localized shape variations. Despite this limitation, the classifier reliably distinguished neutral back configurations, which represent the majority of observed postures.

Overall, these results provide a positive answer to **RQ3**: the proposed classifiers can accurately predict high-level feline posture descriptors in most cases. The observed performance limitations are primarily associated with underrepresented or visually ambiguous classes rather than shortcomings of the underlying geometric modeling, confirming the suitability of the proposed approach for automated feline posture analysis.

### 7.3 End-to-end experiments on videos

**Figure 25:**
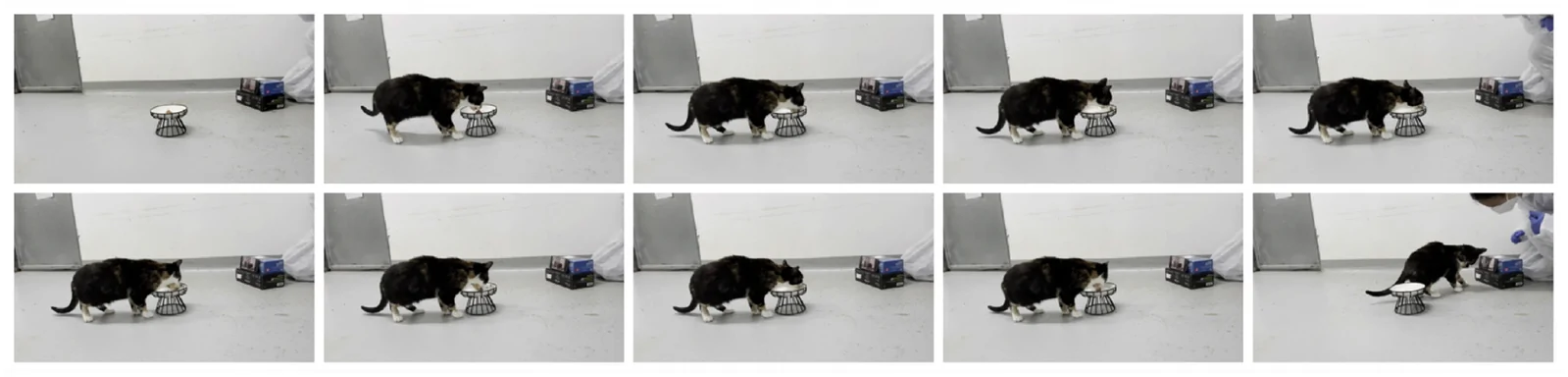
Uniform temporal sampling of *Gioconda* video: 10 frames arranged in a 2 × 5 grid.

**Figure 26:**
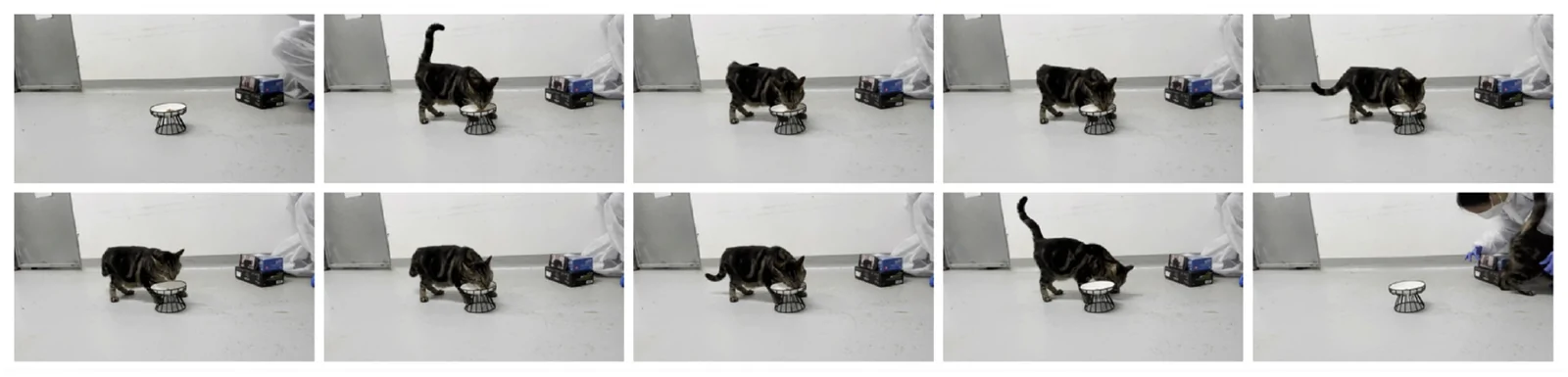
Uniform temporal sampling of *Igor* video: 10 frames arranged in a 2 × 5 grid.

In this section, we evaluate the proposed pipeline in an end-to-end setting using two real-world video sequences recorded during cat palatability tests, in order to address **RQ4** and **RQ5**. These experiments analyze the behavior of the complete pipeline when applied to continuous video streams acquired in a realistic experimental context, from initial detection to final posture classification. The two selected videos, *Gioconda* and *Igor*, were chosen to expose contrasting operational conditions primarily related to differences in the cat's body positioning with respect to the camera and the resulting degree of body-part occlusion. These factors strongly influence the availability of valid side-view frames and the reliability of keypoint and silhouette estimation. To qualitatively illustrate these differences, Figures 25, 26 present a uniform temporal sampling of frames from the *Gioconda* and *Igor* videos, respectively. Rather than aiming for statistical generalization, this analysis is intended as an in-depth, illustrative assessment of the pipeline's robustness and failure modes under realistic conditions, complementing the large-scale quantitative evaluations presented in the previous subsections.

Table 10 summarizes the number of frames retained at each stage of the processing pipeline for both video sequences, providing an empirical answer to **RQ4**. For the *Gioconda* video, a large proportion of frames successfully pass the early detection and model agreement stages, with 87.3% of the total frames retained after YOLO–DensePose agreement. Subsequent posture and view filtering further reduce the frame count, yet 1,487 frames (approximately 46.3% of the original video) remain after all filtering stages, indicating that under stable viewing conditions and sustained standing side-view behavior, the pipeline can process a substantial fraction of frames end to end.

**Table 10:** Frame counts per video at successive stages of the processing pipeline.

	Detection and model agreement			Posture and view filtering		
Video	Total	YOLO	YOLO–DP	Standing	Side view	MMPose–DP + Filt.
Gioconda	3,215	2,808	2,798	2,110	2,024	1,487
Igor	2,001	1,573	1,551	1,044	1,022	42

Total denotes the total number of frames in each video. YOLO corresponds to frames in which a cat is detected by the YOLO object detector. YOLO–DP indicates frames where YOLO detections are consistent with DensePose outputs. Standing refers to frames in which the cat is estimated to be in an upright (standing) posture. Side view denotes frames classified as side-view. MMPose–DP + Filt. corresponds to frames where MMPose and DensePose predictions agree after applying all posture, view, and quality filtering steps. Note that each successive stage only considers the frames that have passed the filtering from the previous column.

In contrast, the *Igor* video exhibits a markedly different behavior: although detection and model agreement stages retain a comparable proportion of frames, the number of frames surviving posture, view, and consistency filtering drops sharply, with only 42 frames (about 2.1% of the total) satisfying all requirements. This substantial reduction is primarily caused by frequent occlusion of the cat's tail and by cases in which the head region is not clearly delineated in the extracted silhouette. These issues arise because the cat is often not perfectly aligned with the camera, resulting in partial self-occlusions and deviations from an ideal side-view configuration. While the view classifier may still predict such frames as side views, the subsequent geometric validation steps reject them due to unreliable keypoint localization or silhouette-based feature extraction. Overall, these results indicate that, in realistic video streams, the proportion of frames that successfully traverse all stages of the pipeline can vary substantially, ranging from a small fraction to nearly half of the video, depending on scene conditions, body orientation, and the degree of body-part occlusion.

To address **RQ5**, Table 11 presents the per-video accuracy of the high-level posture classifiers, computed on frames that successfully passed all stages of the processing pipeline. For both videos, the head pitch and back inclination classifiers achieve consistently high accuracy, exceeding 97% in all cases and reaching near-perfect performance for the *Gioconda* sequence. Similarly, tail configuration classification performs robustly, with accuracy values above 99% for *Gioconda* and perfect classification for *Igor*. These results indicate that, when valid frames are available, the geometric and rule-based classifiers can reliably estimate most posture descriptors in continuous video streams.

**Table 11:** Per-video accuracy of the high-level posture classifiers, computed on frames that successfully passed all detection, posture, view, and consistency filtering stages.

Video	Head pitch	Back inclination	Back curvature	Tail configuration
Gioconda	99.9%	99.2%	18.7%	99.2%
Igor	97.6%	100%	100%	100%

In contrast, back curvature classification exhibits markedly different behavior across the two videos. While perfect accuracy is achieved for the *Igor* video, the accuracy drops substantially for *Gioconda*. This discrepancy is primarily attributable to the limited number of curved-back instances and to the sensitivity of the curvature metric to localized shape variations, as previously discussed in Section 7.2. In the *Gioconda* sequence, subtle posture changes and partial curvature concentrated in specific body regions lead to inflated curvature estimates, resulting in systematic misclassification. This behavior highlights the greater sensitivity of curvature estimation to video-specific posture dynamics and reinforces the need for cautious interpretation of this descriptor in unconstrained settings.

Overall, these results provide a positive answer to **RQ5**: although the successive filtering stages of the pipeline are necessarily rigorous and lead to substantial frame rejection in challenging conditions, the high-level posture classifiers exhibit reliable performance on the frames that successfully traverse the full pipeline. For these retained frames, the proposed end-to-end approach can accurately estimate and classify multiple feline posture descriptors in real-world video sequences. The observed limitations are largely confined to back curvature estimation under specific motion patterns and do not affect the robustness of head pitch, back inclination, or tail configuration analysis. Together with the frame retention analysis presented earlier, these findings demonstrate that the filtering strategy effectively prioritizes reliability over coverage, resulting in accurate posture classification on valid frames while clearly delineating the practical constraints of deploying the proposed pipeline in realistic monitoring scenarios.

The contrast between the two videos also illustrates a practical boundary condition of the current system. When the animal is not consistently aligned with the camera in a standing side-view configuration, as observed in the *Igor* video, the proportion of usable frames can drop to a small fraction of the recording (2.1% in this case). This indicates that, in its present form, the proposed framework is best suited to deployment scenarios in which a fixed camera reliably captures the animal's habitual passage zones in profile, such as feeding stations, doorways, or resting areas, rather than to fully unconstrained, multi-angle monitoring. Mitigating this constraint would likely require complementary strategies such as multiple camera viewpoints or pose-normalization techniques capable of handling partial occlusion and off-axis body orientations, both of which are identified as directions for future work (Section 8).

### 7.4 Behavioral interpretation case study

The previous experiments focused on evaluating the technical performance of the proposed framework, including posture classification accuracy, anatomical consistency validation, frame retention, and end-to-end performance in real-world videos. However, an equally important question is whether the extracted posture descriptors can support meaningful behavioral interpretation in practical applications.

To address **RQ6**, we present two representative case studies based on video recordings obtained during routine feline observations. Although both examples involve interactions with a food bowl, the purpose of this analysis is not to evaluate feeding behavior itself, but rather to demonstrate how temporal patterns of head and tail posture can provide interpretable behavioral context and facilitate the characterization of different behavioral trajectories.

#### 7.4.1 Case A: sustained investigation without behavioral transition

**Figure 27:**
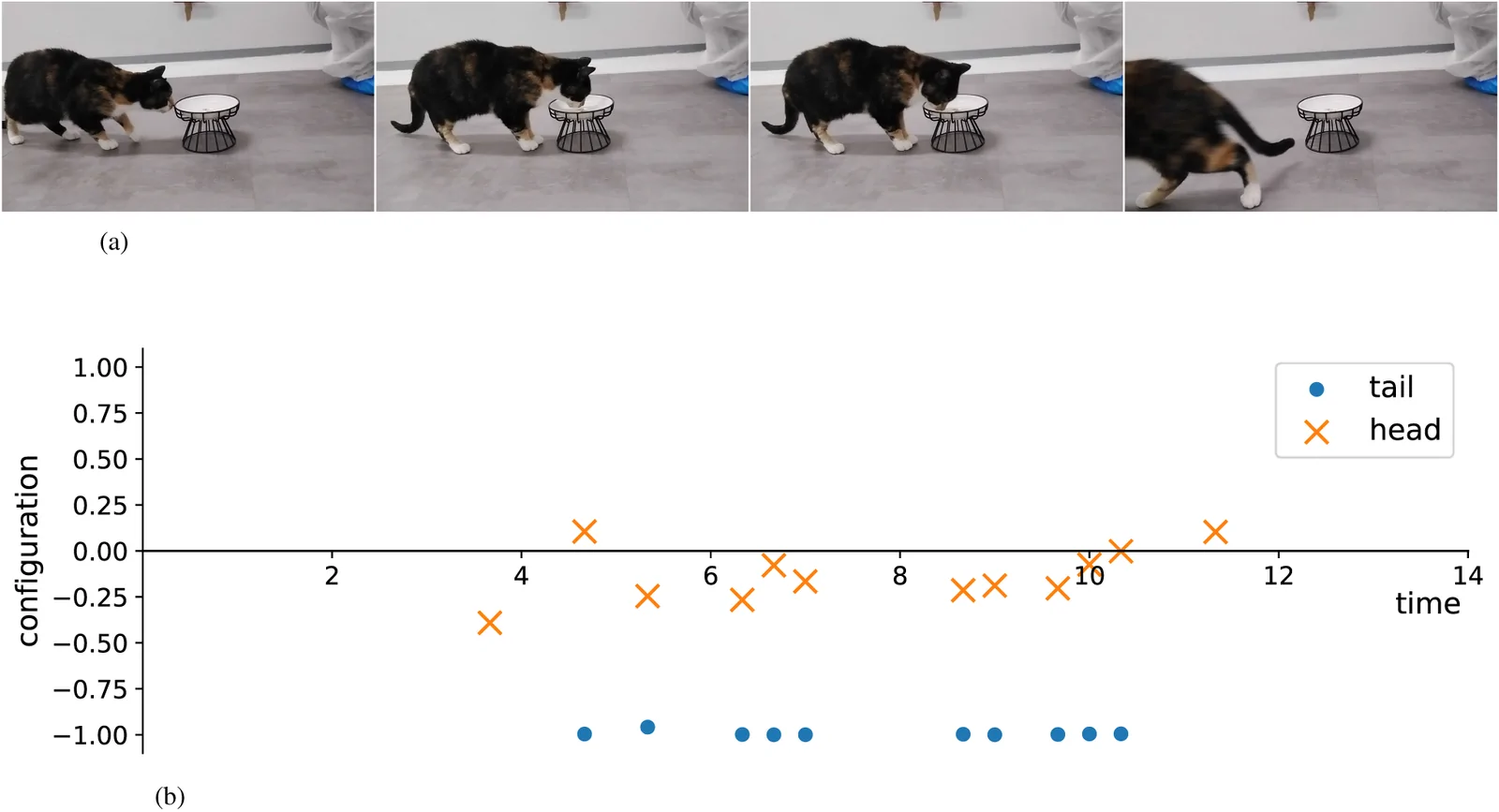
Behavioral interpretation case study (Case A). The upper panel shows uniformly sampled frames from the video, while the lower panel presents the temporal evolution of the posture descriptors generated by the proposed framework. Throughout the observation period, both head and tail remain predominantly in downward configurations, corresponding to a prolonged investigative phase without an observable behavioral transition. **(a)** Uniform temporal sampling of video A. The cat repeatedly investigates the food bowl but does not consume the food. **(b)** Temporal evolution of the automatically extracted head-pitch and tail-configuration descriptors for Case A.

Figure 27 presents uniformly sampled frames from video A together with the corresponding temporal evolution of the automatically extracted head-pitch and tail-configuration descriptors. In this case, the cat repeatedly approached the bowl and engaged in prolonged olfactory investigation of the food. However, no food consumption was observed during the recording period.

The posture descriptors reveal a remarkably stable behavioral pattern throughout the entire observation. Both head pitch and tail configuration remain in the downward state most of the time, indicating a persistent posture profile with little variation over time. From a behavioral perspective, this trajectory is characterized by sustained investigation without transition to a subsequent activity. The cat continuously interacts with the food source, yet the posture descriptors suggest that the behavioral state remains largely unchanged during the observation period.

Importantly, the framework does not determine the underlying cause of this behavior. The persistent low-posture profile may be compatible with several explanations, including cautious investigation, environmental influences, novelty effects, individual temperament, or low food attractiveness. Nevertheless, the descriptors provide information that extends beyond the simple observation that the cat did not eat. They reveal that the interaction remained confined to a prolonged investigative phase and did not evolve toward a different behavioral pattern.

#### 7.4.2 Case B: transition from approach to sustained engagement

**Figure 28:**
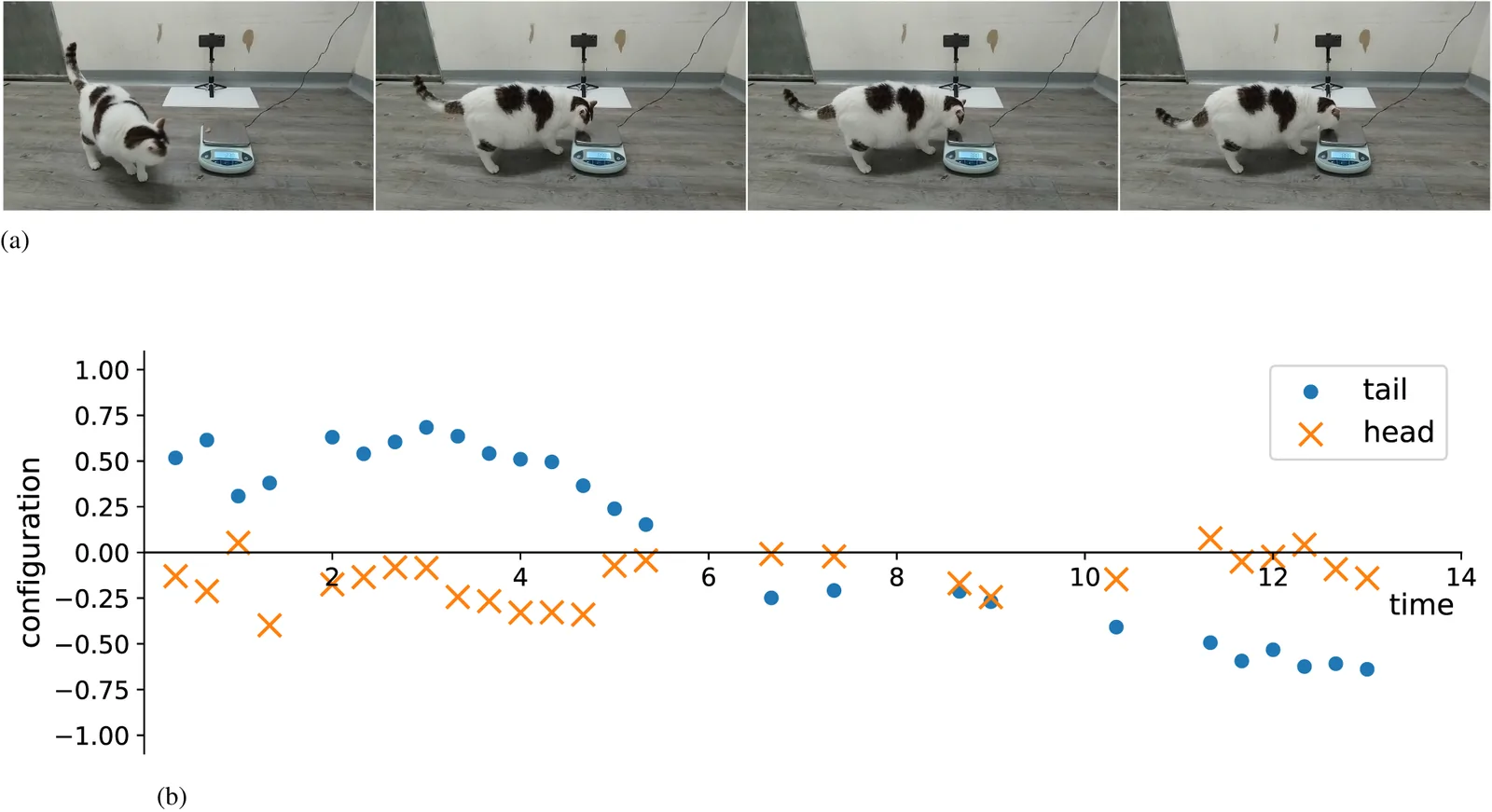
Behavioral interpretation case study (Case B). The upper panel shows uniformly sampled frames from the video, while the lower panel presents the temporal evolution of the posture descriptors generated by the proposed framework. The descriptors reveal a behavioral transition characterized by an initially elevated tail during approach, followed by a gradual shift toward a lower tail configuration during sustained feeding activity. **(a)** Uniform temporal sampling of Case B. The cat approaches the bowl, investigates the food, and subsequently remains engaged in the interaction. **(b)** Temporal evolution of the automatically extracted head-pitch and tail-configuration descriptors for Case B.

Figure 28 presents the second case study. In contrast to Case A, the temporal evolution of the posture descriptors reveals a clear behavioral transition. During the first 4 s, the tail is predominantly classified as upward while the cat approaches the bowl, whereas the head remains directed downward for most of the sequence. After reaching the bowl, the cat investigates the food and subsequently begins eating. Near 6 seconds, the tail gradually transitions from an upward to a downward configuration while feeding continues.

The extracted descriptors therefore reveal a behavioral trajectory that would be difficult to summarize using a single observation alone, distinguishing an initial approach phase from a subsequent feeding phase. It begins with approach and investigation, followed by sustained feeding activity. The elevated tail posture observed during the initial approach is consistent with active engagement with the environment and voluntary interaction with the food source. Once feeding is initiated, the progressive lowering of the tail coincides with a shift from approach and investigation to sustained food consumption. Rather than remaining in a single behavioral state throughout the observation period, the cat exhibits a measurable evolution of posture that can be tracked automatically over time.

The ability to identify such temporal transitions is particularly valuable because they are difficult to quantify objectively through manual observation alone. By converting posture into continuous time-resolved descriptors, the proposed framework enables behavioral evolution to be analyzed and summarized automatically.

#### 7.4.3 Discussion

Taken together, these examples illustrate how the proposed framework can support behavioral interpretation by providing structured descriptions of behavioral trajectories over time. Although both cats approached and investigated the same environmental stimulus, the extracted posture descriptors revealed substantially different temporal patterns. In Case A, the behavior remained confined to a prolonged investigative phase characterized by stable head-down and tail-down postures. In Case B, the behavior evolved through a sequence of postural changes that reflected a transition from approach and investigation to sustained engagement.

These observations demonstrate that the proposed framework provides information beyond static posture classification. By continuously tracking posture descriptors such as head pitch and tail configuration, the system enables behavioral sequences to be objectively characterized, compared, and summarized. While the present study does not establish causal relationships between posture and specific emotional, motivational, or welfare states, the results illustrate how automated posture analysis can generate interpretable behavioral descriptors that may assist veterinarians, animal behavior researchers, and welfare specialists in understanding feline behavior in naturalistic settings.

## 8 Conclusion and future work

This work presented an automated and interpretable framework for feline posture analysis based on the integration of deep learning and geometric modeling. By combining object detection, anatomical segmentation, pose estimation, and rule-based geometric reasoning through a structured processing pipeline, the proposed framework enables detailed and non-invasive characterization of cat posture from side-view RGB images captured with low-cost cameras. The approach systematically decomposes posture into meaningful components, including head orientation, back inclination and curvature, and tail configuration, providing a structured representation of feline body language that supports objective behavioral characterization.

Experimental results demonstrated that the proposed lightweight neural networks reliably detect both overall postures and side-view configurations, achieving accuracies above 92% and outperforming significantly larger VGG19-based models despite using approximately 38 times fewer parameters. In addition, the geometric classifiers showed strong performance for most high level posture descriptors, with particularly robust results for head pitch, back inclination, and tail orientation. These findings confirm that silhouette based and keypoint based geometric features, when carefully validated and combined with deep learning outputs, can effectively capture relevant aspects of feline body language. Although back curvature and neutral tail configurations remain challenging, primarily due to class imbalance and subtle visual differences, the system consistently performs well for the most behaviorally informative and frequently occurring postural states.

A consistent limitation across the geometric classifiers is class imbalance: underrepresented configurations, such as arched back and neutral tail, consistently exhibited lower F1-scores than their majority-class counterparts (Section 7.2.4). This pattern indicates that the current decision thresholds and training data are better calibrated for the most frequently observed postures. Importantly, this limitation stems primarily from the composition of the training data available for the present study rather than from an intrinsic constraint of the underlying geometric features, and classifier performance for these configurations would be expected to improve with a training set containing a larger and more representative sample of the minority classes. Expanding and rebalancing the dataset, through targeted data collection or augmentation for underrepresented postures, therefore remains a priority for improving the reliability of the framework across the full range of feline postures.

A further limitation concerns the generalizability of the framework. Although the dataset comprised more than 30 individual, non-pedigree domestic shorthair cats with diverse coat colors and patterns (Section 3), all data were collected at a single research facility under controlled palatability-testing conditions. Consequently, the reported accuracies should not be assumed to generalize directly to other breeds, body conformations, or the wider range of environmental and lighting conditions encountered in domestic, shelter, or clinical settings. Broader, multi-site data collection is required before general deployment claims can be made.

End-to-end evaluations on real-world video sequences highlighted both the strengths and practical limitations of the processing pipeline. While strict filtering stages necessarily reduce the number of usable frames under challenging viewing conditions, the retained frames yield highly accurate posture classifications. This design choice prioritizes reliability and interpretability, making the system suitable for applications where precision is more critical than continuous coverage.

The behavioral interpretation case studies further demonstrated the practical value of the proposed descriptors. By continuously tracking posture features such as head pitch and tail configuration, the framework can characterize behavioral trajectories and reveal temporal patterns that are not captured by simple outcome-based observations alone. Although the present study does not establish causal relationships between posture and specific emotional, motivational, or welfare states, the results illustrate how automated posture analysis can provide objective and interpretable behavioral context for veterinary, welfare, and research applications.

Overall, this study contributes a scalable and modular solution for automated feline posture assessment, with clear potential applications in animal welfare monitoring, veterinary behavioral medicine, and behavioral research. Beyond accurate posture classification, the proposed framework generates continuous and interpretable posture descriptors that can support the objective characterization of feline behavior over time. These capabilities highlight the potential of computer vision systems to augment human observation and provide standardized behavioral measurements in both research and practical settings.

Future work will focus on expanding and balancing the dataset, improving robustness to occlusions and non-ideal viewpoints, and validating the relationship between the proposed posture descriptors and established feline pain, stress, welfare, and behavioral assessment instruments. Longitudinal studies will also be necessary to determine how temporal posture trajectories can support the automated identification of clinically relevant behavioral patterns in real-world environments. In addition, future investigations should evaluate the influence of camera characteristics, including higher-quality RGB sensors and different acquisition settings, on frame retention and posture descriptor accuracy. Such developments may ultimately enable the integration of automated posture analysis into decision-support systems for veterinary behavioral assessment and animal welfare monitoring.

## Data Availability

The data analyzed in this study is subject to the following licenses/restrictions: The datasets used in this study are not publicly available and are not available upon request. The data are proprietary to CEVA Santé Animale and are subject to confidentiality and contractual restrictions that prohibit external sharing. Requests to access these datasets should be directed to thales@ic.ufal.br.

## Footnotes

1
https://github.com/facebookresearch/detectron2/blob/main/projects/DensePose/configs/evolution/densepose_R_50_FPN_DL_WC1M_3x_Atop10P_CA_B_finesegm.yaml
